# The French Connection: The First Large Population-Based Contact Survey in France Relevant for the Spread of Infectious Diseases

**DOI:** 10.1371/journal.pone.0133203

**Published:** 2015-07-15

**Authors:** Guillaume Béraud, Sabine Kazmercziak, Philippe Beutels, Daniel Levy-Bruhl, Xavier Lenne, Nathalie Mielcarek, Yazdan Yazdanpanah, Pierre-Yves Boëlle, Niel Hens, Benoit Dervaux

**Affiliations:** 1 Médecine Interne et Maladies Infectieuses, Centre Hospitalier de Poitiers, Poitiers, France; 2 EA2694, Université Droit et Santé Lille 2, Lille, France; 3 Interuniversity Institute for Biostatistics and statistical Bioinformatics, Hasselt University, Hasselt, Belgium; 4 CRESGE, Université Catholique de Lille, Lille, France; 5 Centre for Health Economics Research & Modeling Infectious Diseases (CHERMID), Vaccine & Infectious Disease Institute, Universiteit Antwerpen, Antwerpen, Belgium; 6 Département des maladies infectieuses, InVS, Saint-Maurice, France; 7 Département d’information médicale, Université de Lille Nord de France, Centre Hospitalier Universitaire de Lille, Lille, France; 8 Center for Infection and Immunity of Lille, Institut Pasteur de Lille, Lille, France; 9 INSERM U1019, Lille, France; 10 CNRS UMR8204, Lille, France; 11 Université Lille-Nord de France, Lille, France; 12 Service des Maladies Infectieuses et tropicales, Hôpital Bichat Claude Bernard, Paris, France; 13 UMR-S 707, INSERM, Paris, France; 14 DRCI, Centre Hospitalier Universitaire de Lille, Lille, France; Centers for Disease Control, TAIWAN

## Abstract

**Background:**

Empirical social contact patterns are essential to understand the spread of infectious diseases. To date, no such data existed for France. Although infectious diseases are frequently seasonal, the temporal variation of contact patterns has not been documented hitherto.

**Methods:**

COMES-F is the first French large-scale population survey, carried out over 3 different periods (February-March, April, April-May) with some participants common to the first and the last period. Participants described their contacts for 2 consecutive days, and reported separately on professional contacts when typically over 20 per day.

**Results:**

2033 participants reported 38 881 contacts (weighted median [first quartile-third quartile]: 8[5–14] per day), and 54 378 contacts with supplementary professional contacts (9[5–17]). Contrary to age, gender, household size, holidays, weekend and occupation, period of the year had little influence on the number of contacts or the mixing patterns. Contact patterns were highly assortative with age, irrespective of the location of the contact, and gender, with women having 8% more contacts than men. Although most contacts occurred at home and at school, the inclusion of professional contacts modified the structure of the mixing patterns. Holidays and weekends reduced dramatically the number of contacts, and as proxies for school closure, reduced R_0_ by 33% and 28%, respectively. Thus, school closures could have an important impact on the spread of close contact infections in France.

**Conclusions:**

Despite no clear evidence for temporal variation, trends suggest that more studies are needed. Age and gender were found important determinants of the mixing patterns. Gender differences in mixing patterns might help explain gender differences in the epidemiology of infectious diseases.

## Introduction

Mathematical modelling of infectious diseases is invaluable to evaluate control and prevention strategies by comparing their (cost-)effectiveness and to inform public health decision makers. While most models make assumptions on transmission parameters, social contact data studies estimate the probability of contacts between individuals, and consequently of potential pathogen transmission. For instance, social contact data studies have shown better goodness-of-fit than mathematical and parsimonious models on seroprevalence data for varicella[1]. Contact diaries have several advantages in measuring of the frequency and intensity of contacts between individuals. They are easy to use, capture social interaction in a wide range of settings and do not rely on peer-group participants[2]. They successfully explained age-specific patterns of infection such as varicella-zoster virus, parvovirus B19[3], mumps[4], influenza[4] and pertussis[5]. Nonetheless, defining a contact suitable for infectious disease transmission remains difficult and varies according to pathogen[2,6]. A population-based contact survey provides the basic material allowing to build contact matrices with different levels of contact intimacy (e.g. physical or/and long-duration contacts versus conversational or/and short-duration contacts).

Focusing on 8 European countries, POLYMOD was the first large-scale study to report on contacts between individuals[7]. To date, no such data existed for France. Fumanelli et al[8] estimated contact matrices by inferring the structure of social contacts from demographic data, but at the expense of substantial differences with the empirical contact matrices from the POLYMOD study. Time-Use surveys are widely available and provide a valuable alternative to estimate contact matrices, but they are often restricted to participants older than 8 years[6,9]. With regards to the pandemic influenza A/H1N1 virus, a French household-based survey reported meetings made by participants but with information restricted to the place and the age distribution of contacts[10].

Seasonality is a common feature in infectious diseases, usually attributed to environmental factors such as temperature or humidity[11]. Term-time forcing for measles[12] and other childhood infections[13] suggests the importance of behavioural factors. But few studies have evaluated change in the number of contacts for given persons over a period of time[14,15]. None have compared change in mixing patterns overtime. Hence, while contact matrices have been developed at the country-wide level, they lack in temporal information.

In this paper we describe the first large-scale population survey investigating contact patterns in France and their temporal variations. Taking advantage of the natural heterogeneity of France–one of the largest European countries- and using one of the largest sample sizes for a country-based population survey carried out to date, we have estimated French contact rates. We have reassessed the influence on mixing patterns of weekends and holidays as well as gender, children’s contact patterns or class size. We have also explored the influence of people with high numbers of professional contacts on one or two consecutive days.

## Methods

### Study design

**Fig 1:**
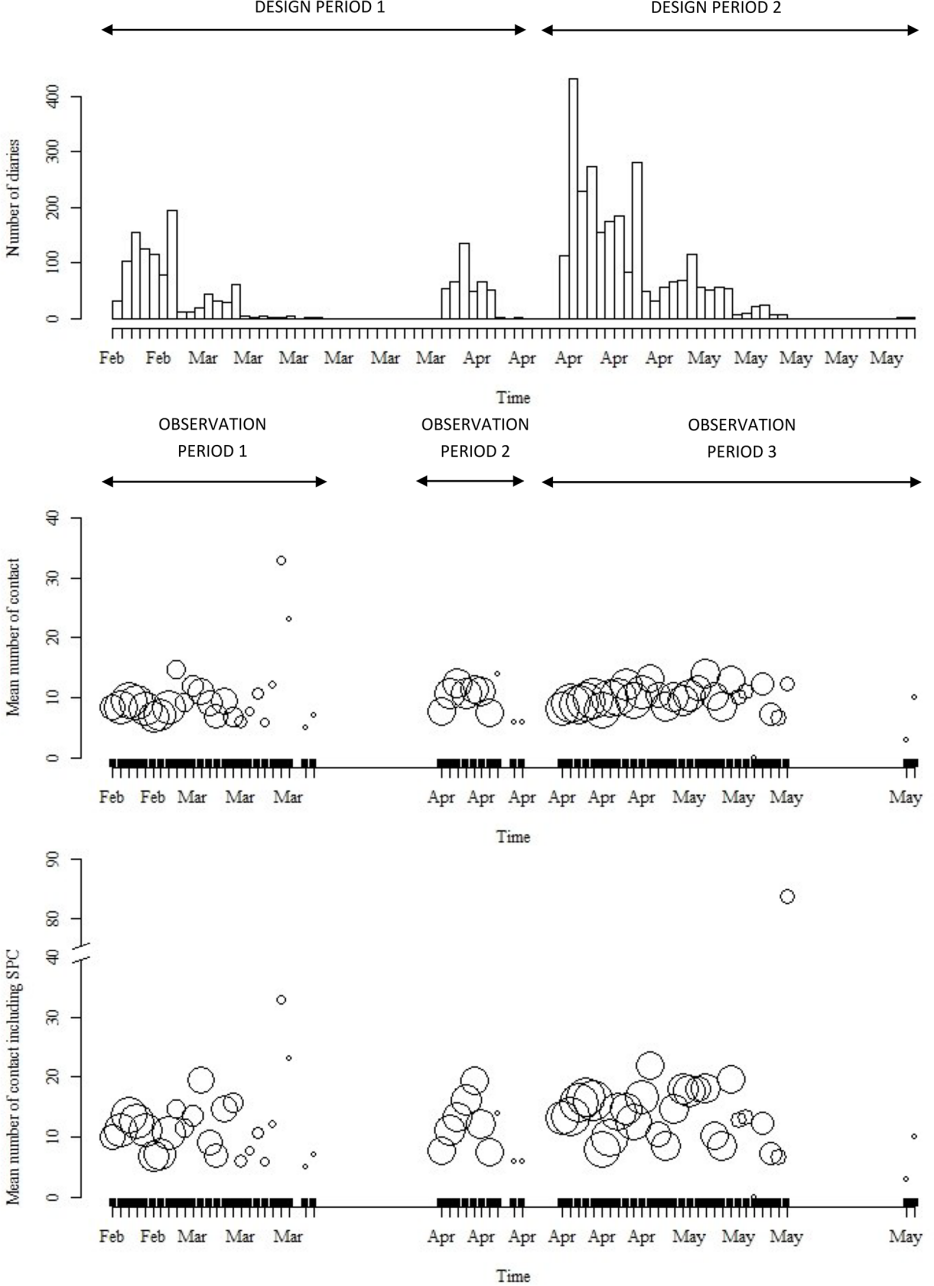
Timeline of the study, showing the distribution of participants and contacts over time. The periods of inclusion were February, 20^th^–March,17^th^; April,1^st^–April, 7^th^; April,16^th^–May, 14^th^. The dot size is proportional to the log of participant’s number. (Design Period 1: 34 days; Design Period 2: 29 days)

The study population was randomly sampled from all over France (excluding overseas territories) and planned over 2 time periods (February-March/April-May 2012) (Fig 1). An oversight leading to recruit fewer participants than originally planned during the February-March period (Actual Period 1), an additional period (Actual Period 2) was added in April to complete “Design Period 1”. Recruitment during “Design Period 2” was completed according to plan (Actual Period 3). The Actual Period 2 being chronologically close to “Design Period 2”, we presented data analyses according to (1) Design Periods 1 and 2, (2) the 3 Actual Periods 1, 2 and 3 and (3) to Actual Period 1 and a combination of Actual Periods 2 and 3. Winter holidays lasted 2 weeks at different dates in February-March according to the place of residency, while Spring holidays lasted 2 weeks in April-May.

Participants were recruited according to quota for age, gender (sex-ratio = 1), days of the week and school holidays from 24 250 persons contacted by random digit dialling (landlines and mobile numbers). Diaries (S1 Fig) were sent to 3977 people who have accepted to participate, among whom 2033 actually participated (729 during Design Period 1; 1304 during Designed Period 2). Participants common to Design Periods 1 & 2 (n = 278) represented respectively 38% and 21% of participants. Only one person per household could participate. Children and teenagers were oversampled to gain accuracy on age groups known to contribute largely to the spread of infections.

Participants had to describe their environment (household, workplace, school…), their socio-professional background, and all their contacts for 2 consecutive days on a paper diary. A contact was defined as talking to someone within a distance of less than 2 meters, or skin-to-skin touching. Each contact had to be described with age (or estimated age category), gender, location, frequency, type (skin contact or not) and duration of the contact. A contact was to be reported only once daily in the diary.

The diary was derived from the POLYMOD study but had some additional features. The daily number of potentially recorded contacts was limited to 40 (versus 29 to 90 in POLYMOD[7]). A specific diary for children 0–15 years old (27.9% of all the diaries) was designed with instructions for caregivers to help complete it. This diary had specific questions about childcare, school and location of contacts (school and day care centre).

Participants were coached by phone and could seek information to complete the diary through a hotline and an email address. They were contacted up to 3 times if the diary was not returned. Participants who returned the diary were offered 5€ for themselves or for donation. Participants provided a verbal consent when they accepted to participate in the study, as they were contacted by phone. Moreover, they confirmed their consent by returning the diary. Thus, returning the diary was considered as a written consent. For children younger than 15 years, their consent and the consent of a parent or legal caregiver had to be obtained, in a similar ways (first by phone, then by returning the diary). Children between 15 and 17 were considered as adults, thus, their consent was obtained without requiring an adult. Records were anonymized before analysis, thus identifying participant information was and is not available. The study protocol, as well as the consent procedure, was declared and approved by the French Institutional Review Board correspondent at the Institut Catholique de Lille.

### Data analysis

Data analysis was done using the statistical programming language R 3.1.0. Sampling weights were calculated according to participants’ age, household size (2009 national census, INSEE[16]), weekdays and weekends, regular and holiday periods. Continuous variables are expressed as weighted median (first quartile-third quartile).

#### Number of contacts

Regarding the number of contacts, variable selection was done using random forests[17] (R package randomForest). Age was transformed into five-year age categories and days of the week were transformed into weekday/weekend for data sparseness and model interpretability. Generalized Estimation Equation (GEE)(R package geepack) with a negative binomial distribution were used to regress the number of contacts and the selected variables. Variables influencing the number of contacts were compared with percentage of change and 95% confidence interval (95%CI) based on the estimates from GEE. The GEE approach can handle correlations between repeated observations from the same participants. The degree distribution of the number of contacts was modelled using Generalized Additive Models (GAM) with spline smoothing (R package mgcv[18]) stratified according to age, gender, weekdays and weekend, regular and holiday periods.

#### Who mixes with whom?

Contact matrices were obtained using GAM assuming a negative binomial distribution, using a one-year age interval and a tensor product spline as smooth interaction term between contact age and participant age. Different matrices were calculated: a base-case matrix without supplementary professional contacts, a matrix with physical contacts only, a matrix with supplementary professional contact information and matrices according to the 3 actual periods of the study.

To assess the influence of gender, 2-by-2 matrices were built according to gender and age (≤18 years; >18 years) of both participants and contacts.

To assess the influence of the place of contact, location-based matrices were built using 6 age categories and no smoothing.

Data sparseness prompted us to use different methods to obtain the matrices as described above. The reciprocal nature of contacts was taken into account by a ‘smooth-then-constrain’ approach[19], except for location matrices where no reciprocity was imposed.

Comparing the impact of different mixing patterns on the spread of infectious diseases, we calculated the relative change in the basic reproduction number R0 for a generic epidemic by calculating the ratio of dominant eigenvalues of the respective next generation matrices[20,21] (S1 Text.). Similarly, we used the leading right eigenvector of the specific next generation matrices to calculate relative incidence by age. For the location matrices, we used the eigenvalue of the contact rate matrices as there was no population size by location, warranting a somewhat different interpretation. Resampling was done to estimate 95%CI of leading eigenvalues and eigenvectors. Changes in R0 and contact rate were compared with a ratio and 95%CI based on a non-parametric bootstrap.

#### Professional contacts

Participants with more than 20 daily professional contacts were asked not to report them but rather to provide their total number and age distribution (0–3 years; 3–10 years; 11–17 years; 18–64 years; 64+ years) (referred as “supplementary professional contacts” or “SPC” in the remainder of the paper).

Other contact characteristics were imputed by resampling the characteristics of professional contacts from participants who had between 10 and 20 professional contacts (S2 Text.). We repeated our analyses including the SPC and applied censoring, once at 134 contacts (the 95% percentile) to limit the impact of outliers, and once modelling censor at 29 contacts (S1 Table. Factors influencing the number of contacts, with censoring at 29 similarly to Mossong et al 2008 (non-linear model)), similarly to what had been done in the POLYMOD study. Unless mentioned otherwise, results concerned the model without SPC.

Contact matrices as well as all the data necessary to reproduce our analysis are freely available on www.contactmatrix.fr and on a public figshare repository at http://dx.doi.org/10.6084/m9.figshare.1466917.

## Results

Participants’ age was 37 (19–59) years old, with 46 (2%) <1 year and 795 (39%) <18 years. Women represented 1136 (56%) of participants. According to the designed quotas, 890 (44%) participants had a weekend day among the 2 consecutive participation days, which was always associated with a weekday. Participation days were neither a weekend day nor holiday for 552 (27%) participants. Participants were on average significantly older than the originally recruited people who didn’t send in their diaries (37.1(27.0)y vs 30.9(25.4)y; p<0.001). Non-participants were mostly aged 18–39 years and 3–9 years (whose diaries were filled by an adult, usually aged 18–39 years). Results have to be interpreted within that context (S3 Text. Design issues). Similarly, employment and school enrolment rates should be interpreted with respect to age quotas (S2 Table).

### Number of Contacts

**Fig 2:**
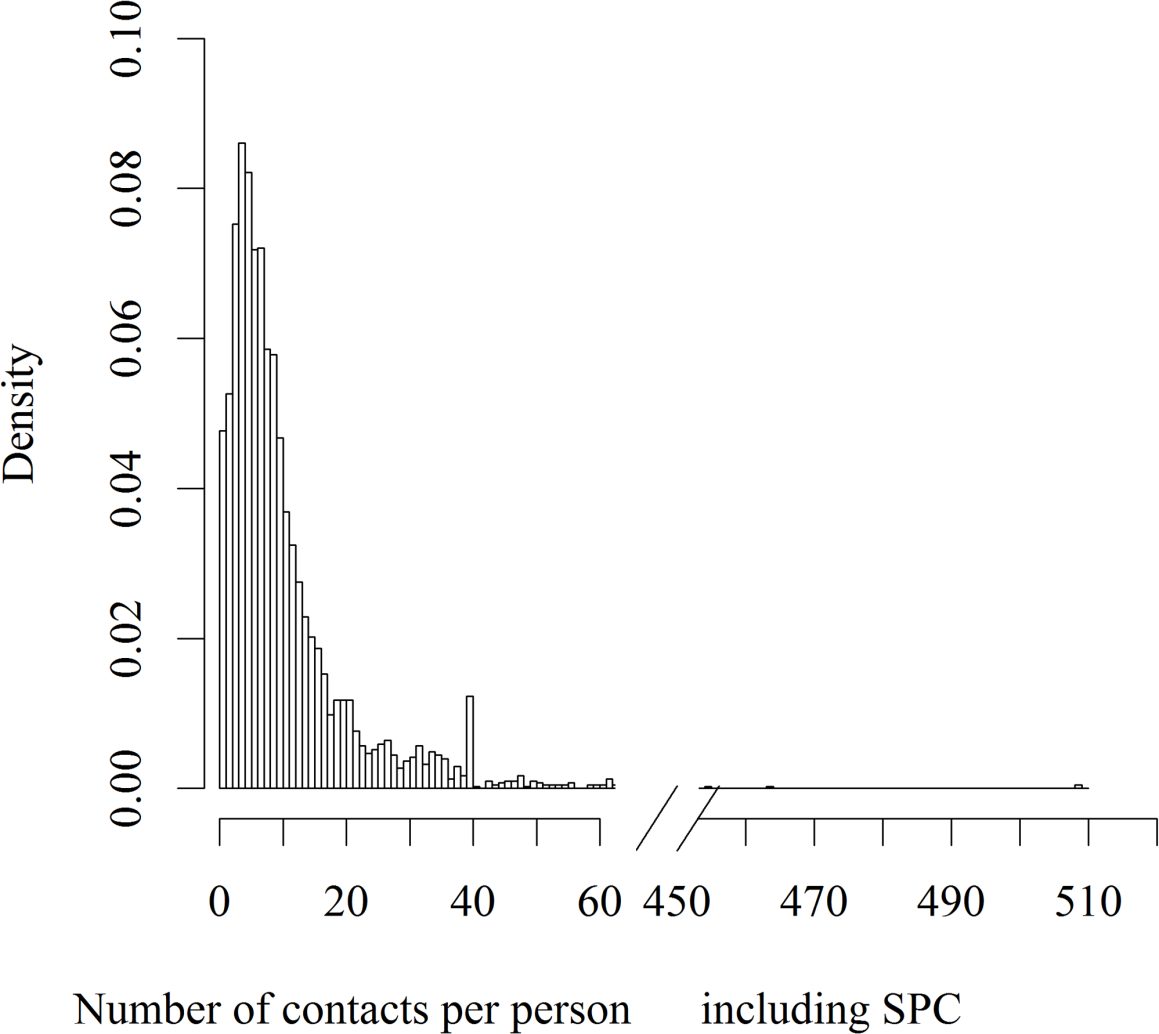
Contact number density. Histogram of the contact number, including SPC (Supplementary Professional Contacts). Limitation at 40 contacts per day explains the peak at 40 contacts.

Participants reported 38 881 contacts (8(5–14) per day; Fig 2), with +9% [1%;18%] more contacts in Design Period 2 but without significant differences between Actual Periods 1, 2 or 3 (Table 1) (S2 Fig), or between the combination of Actual Periods 2 & 3 vs. Actual Period 1 (+4%[-4%;13%]). Factors influencing the number of contacts are summarized in Table 1. Region of residency did not influence the number of contacts.

**Table 1:** Factors influencing the number of contacts (SPC: Supplementary Professional Contacts).

Covariate		Number of participants	Mean (SD) of Number of Reported Contacts	Relative Number of Reported Contacts (95% CI)	Relative Number with SPC (95% CI)	Relative Number with censored SPC (95% CI)
Age (y)	0–4	305	8.64 (7.23)	1	1	1
	5–9	262	10.50 (8.07)	1.31 (1.17–1.47)	1.26 (1.09–1.45)	1.30 (1.15–1.48)
	10–14	160	12.92 (10.44)	1.67 (1.45–1.93)	1.46 (1.22–1.76)	1.54 (1.33–1.80)
	15–19	131	12.96 (9.55)	1.62 (1.41–1.87)	1.48 (1.24–1.77)	1.54 (1.32–1.79)
	20–24	114	11.14 (8.91)	1.74 (1.44–2.11)	1.82 (1.45–2.30)	1.75 (1.42–2.15)
	25–34	108	9.95 (6.70)	1.77 (1.36–2.30)	1.70 (0.86–3.38)	1.37 (0.92–2.03)
	35–44	108	9.93 (7.10)	1.61 (1.24–2.10)	1.42 (0.91–2.21)	1.51 (1.04–2.17)
	45–64	426	9.24 (7.23)	1.86 (1.45–2.40)	1.22 (0.80–1.85)	1.29 (0.92–1.82)
	65+	419	7.01 (5.73)	1.71 (1.32–2.22)	1.27 (0.78–2.06)	1.23 (0.87–1.74)
Gender	Female	1136	9.78 (8.04)	1	1	1
	Male	897	9.29 (7.52)	0.92 (0.86–0.99)	0.80 (0.63–1.02)	0.84 (0.73–0.96)
Household size	1	323	7.88 (6.60)	1	1	1
	2	668	8.14 (6.60)	1.04 (0.94–1.16)	1.03 (0.79–1.33)	0.95 (0.76–1.18)
	3	321	10.49 (8.50)	1.27 (1.12–1.45)	1.11 (0.86–1.43)	1.12 (0.87–1.43)
	4	468	10.99 (8.62)	1.42 (1.25–1.62)	1.28 (0.88–1.86)	1.14 (0.89–1.47)
	5+	253	11.66 (8.61)	1.39 (1.19–1.62)	1.78 (0.92–3.42)	1.37 (0.98–1.93)
Day of the week	Week Day	1584	9.90 (8.13)	1	1	1
	Week End	449	8.37 (6.50)	0.79 (0.73–0.86)	0.44 (0.37–0.52)	0.48 (0.42–0.54)
Participating day	First	1016	9.87 (7.92)	1	1	1
	Second	1016	9.26 (7.72)	0.94 (0.89–0.98)	0.94 (0.87–1.03)	0.96 (0.91–1.02)
Holiday	Regular Day	972	11.05 (9.05)	1	1	1
	Holiday	1061	8.20 (6.20)	0.79 (0.74–0.84)	0.85 (0.66–1.10)	0.85 (0.74–0.99)
Occupation	Under education	909	10.82 (8.80)	1	1	1
	Employed	435	10.67 (7.46)	0.85 (0.68–1.07)	2.64 (1.83–3.80)	2.31 (1.72–3.10)
	Unemployed	685	7.20 (5.89)	0.63 (0.50–0.79)	0.79 (0.56–1.10)	0.76 (0.57–1.02)
Period	Period 1	517	8.74 (7.06)	1	1	1
	Period 2	212	10.27 (8.42)	0.94 (0.82–1.07)	0.93 (0.68–1.27)	0.97 (0.75–1.25)
	Period 3	1304	9.77 (7.98)	1.06 (0.98–1.16)	1.22 (1.03–1.45)	1.15 (0.99–1.33)

**Fig 3:**
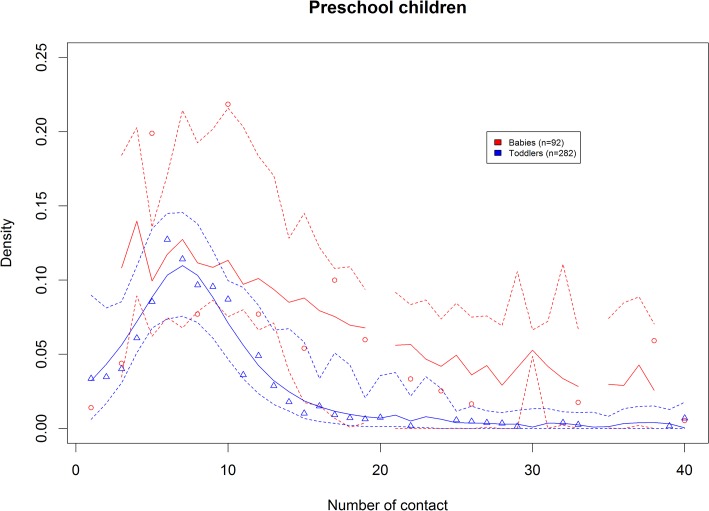
Degree distribution of children <4y, comparing number of contacts between <1y to 1–3y, with density of number of contact. Similar graph with frequency of number of contact is provided as supplementary material S3 Fig.

The relative number of contacts rapidly increased with age to reach a plateau at around 20 years old. Among children, babies (<1 year) had slightly fewer contacts than toddlers (1–3 years) (Fig 3).

**Fig 4:**
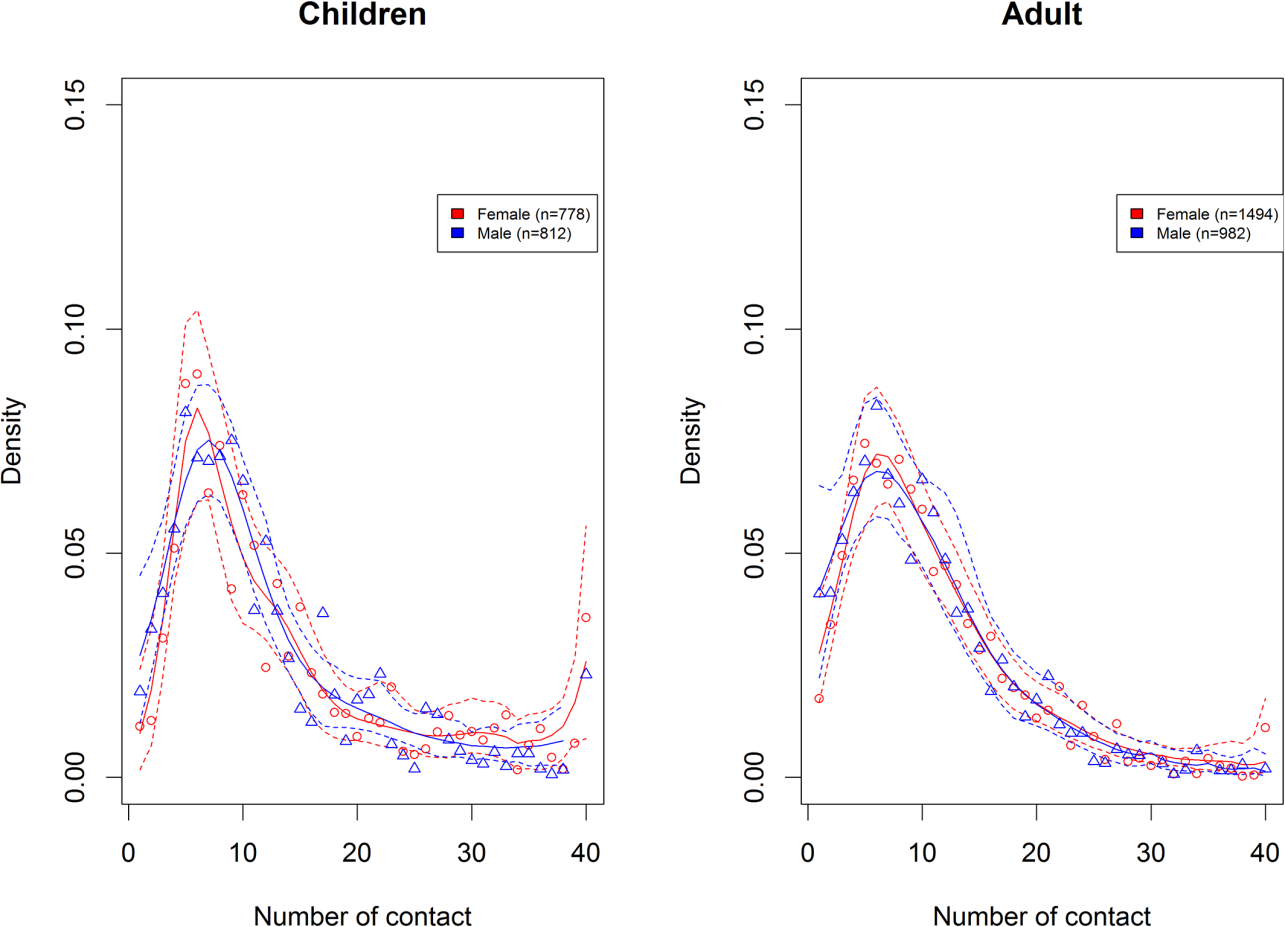
Degree distribution comparing number of contacts according to gender in <18y and >18y, with density of number of contact. Similar graph with frequency of number of contact is provided as supplementary material S3 Fig.

Women had 8% [1%;14%] more contacts than men, mainly due to differences for adult women (Fig 4).

**Fig 5:**
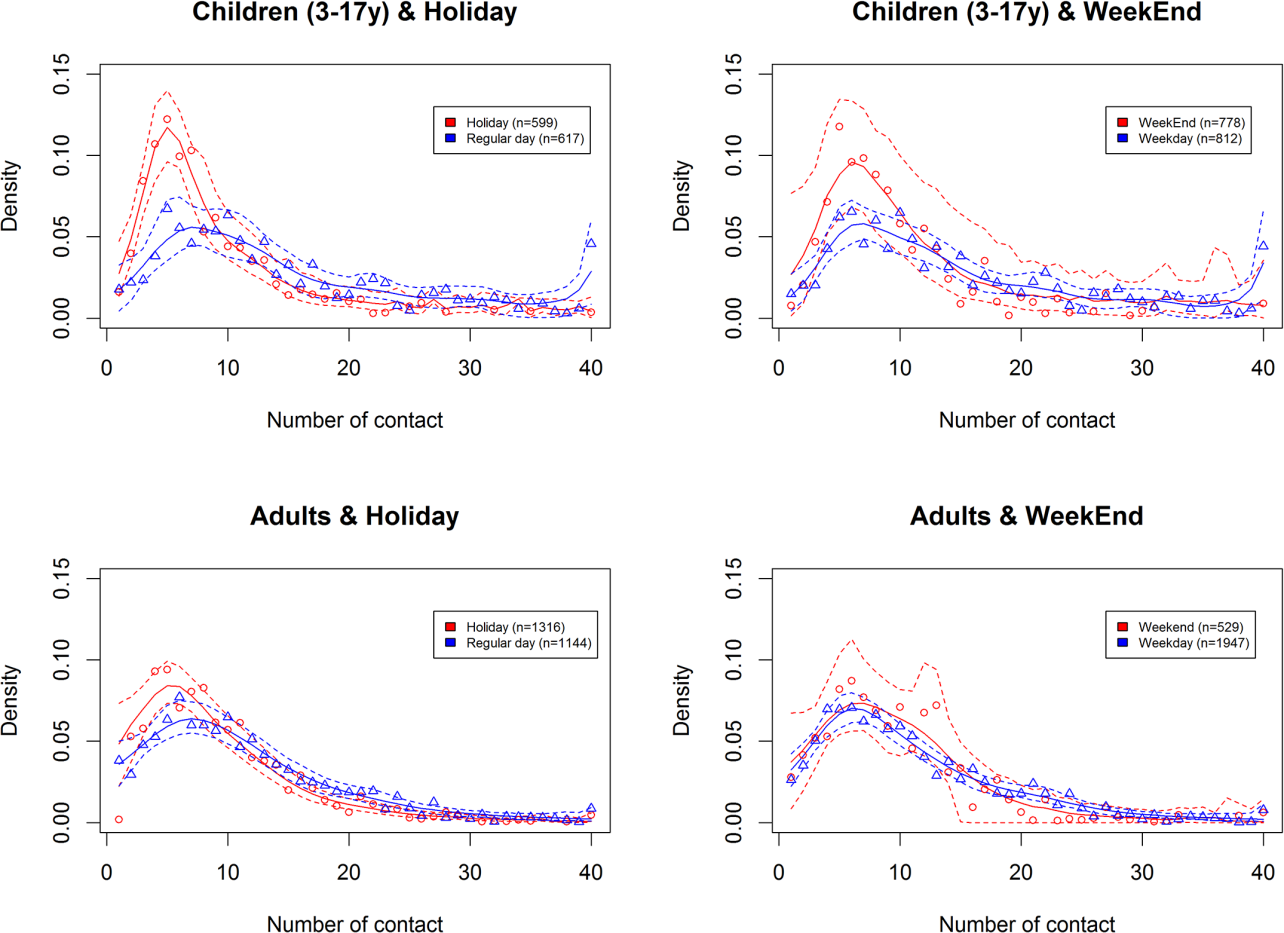
Degree distribution comparing number of contacts according to weekends and holidays in children (3–18y) and adults, with density of number of contact. Similar graph with frequency of number of contact is provided as supplementary material S3 Fig.

During weekends and holidays the number of all contacts decreased respectively by 21% [14%;27%] and 21% [16%;26%] (Table 1), and by 16% [8%;23%] and 19% [13%;25%] for physical contacts The impact was different between children and adults (Fig 5).

**Fig 6:**
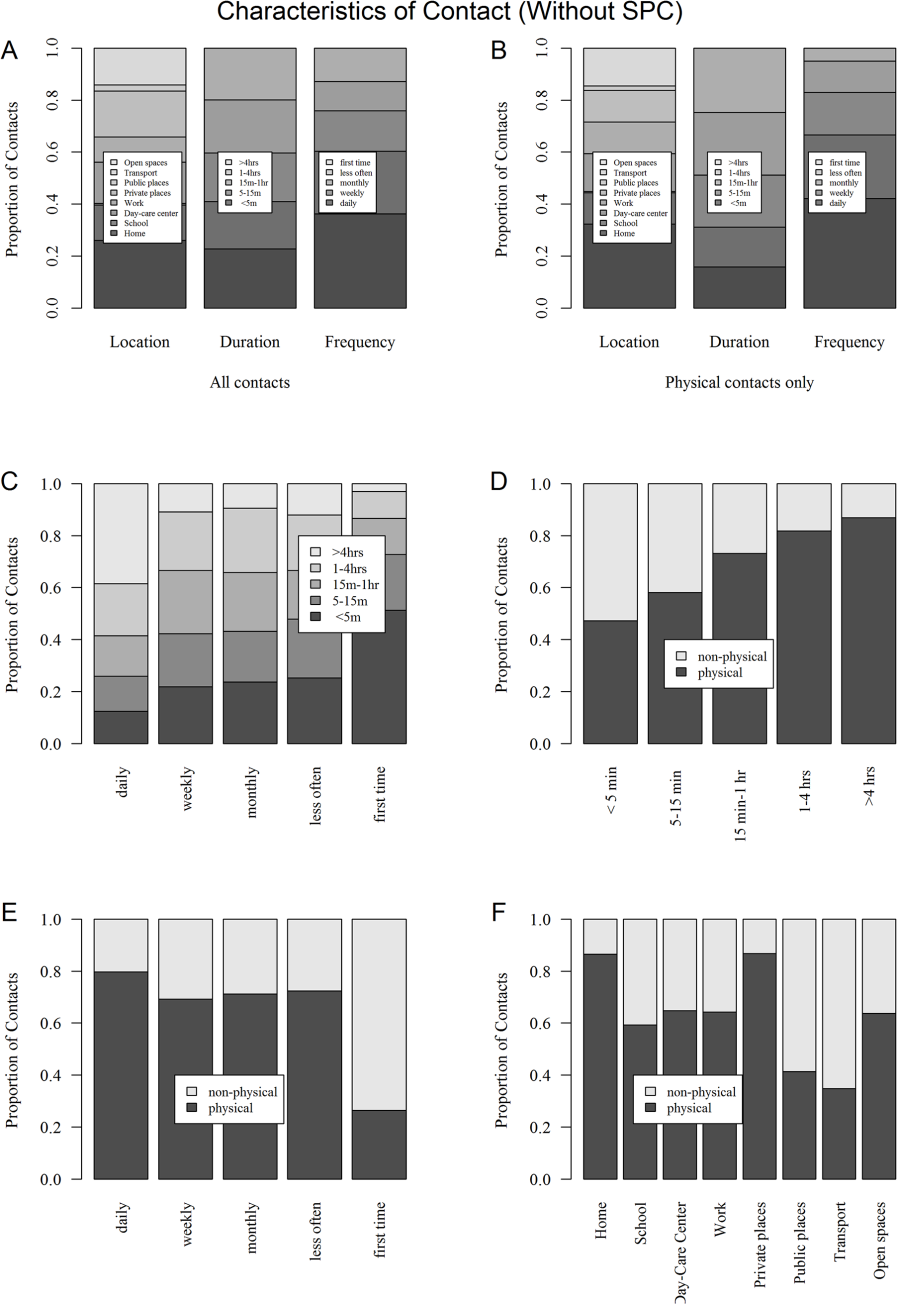
Characteristics of contact (without SPC). Distribution of location, duration and frequency for all contacts (A) and physical contacts (B). Duration of contact according to frequency (C). Proportion of physical contacts according to duration (D), frequency (E) and location (F).

Duration was associated with frequency of contacts, as daily contacts lasted longer than less frequent contacts (Fig 6A & 6C). Physical contacts were associated with longer duration (Fig 6B & 6D) and more frequent contacts (Fig 6E). Physical contacts occurred more often at home or in private places than at work or study place, and rarely during transport (Fig 6D & 6F).

Transportation modes did not significantly influence the number of contacts, despite a trend for higher number of contacts with public transport (+36%[-20%;+131%]).

For a subanalysis of contacts made by participants common to Design Periods 1 & 2, trends were similar as observed in the full data analysis, though only age, household size, regular or holiday period and occupation remained significant.

No association was found between the size of classroom or childcare centre and the total number of contacts or those specifically at kindergarten (4.8[0.0;9.0]), at school (5.0[0.0;16.0]) or study place (6.0[0.0;16.0]).

Participants reported 6%[1%;10%] fewer contacts on the 2nd day of the study. The more contacts they reported on the first day, the larger the proportional decrease in contacts on the second day.

### Who mixes with whom

**Fig 7:**
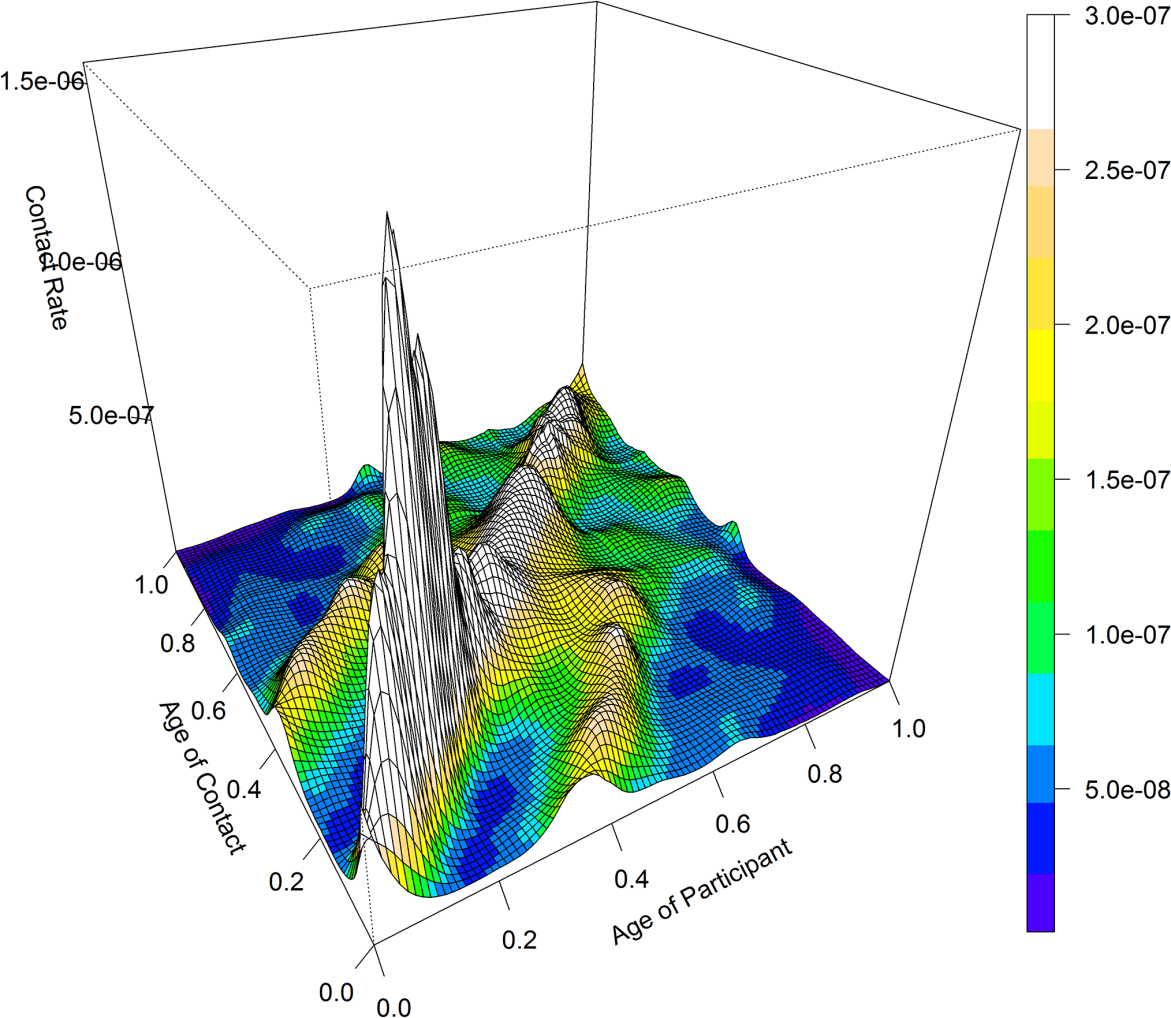
3D representation of the base-case matrix without SPC (Supplementary Professional Contacts).

**Fig 8:**
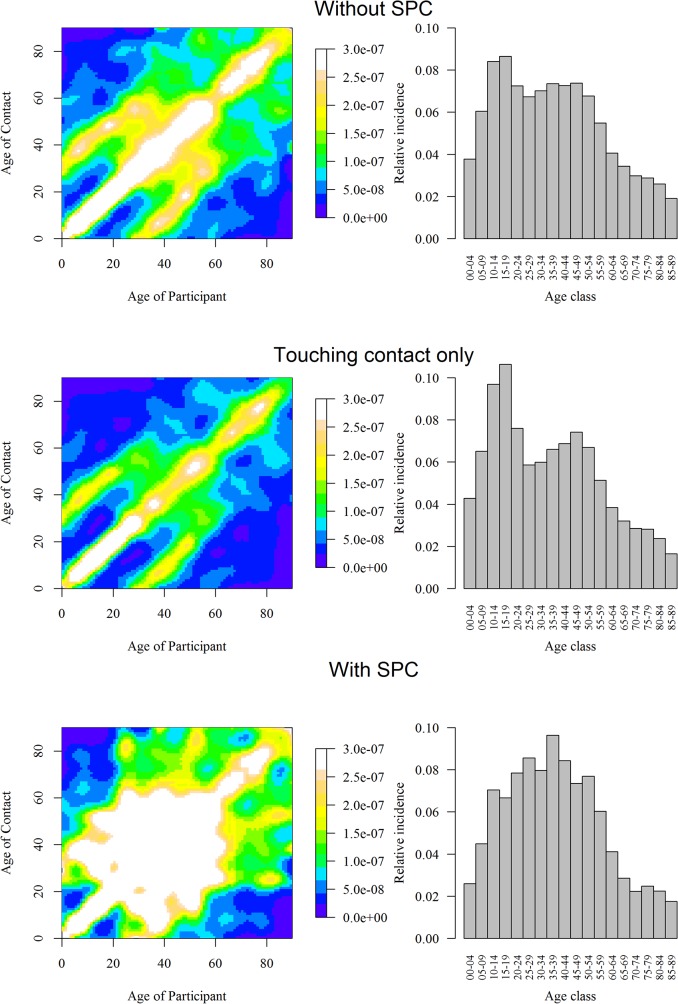
Smoothed contact matrices without SPC, for physical contacts only and with SPC(Supplementary Professional Contacts) (right). Relative incidence of a new emerging infection in a completely susceptible population estimated from the matrix in regard (left).

The R0 of an epidemic occurring during Design Period 2 was 12%[1%;23%] higher than during Design Period 1, but lost significance when comparing 2 out of 3 Actual Periods (P1 vs P2: +13%[-9%;46%]; P1 vs P3: 11%[-4%;26%]). Mixing patterns (Figs 7–8)(S3 Table) also showed an important contribution during the initial phase of an epidemic for the 10–20 year olds (predominantly for Actuals Periods 2 and 3), and for the 35–50 year olds (predominantly for Actual Period 1).

**Fig 9:**
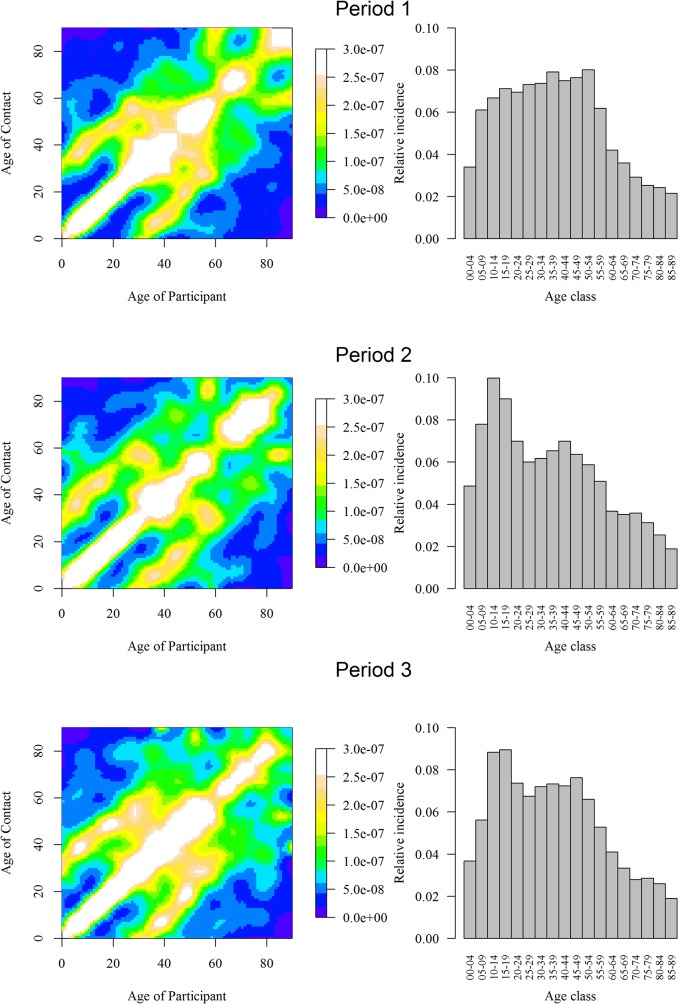
Smoothed contact matrices according to Actual Period (right). Relative incidence of a new emerging infection in a completely susceptible population estimated from the matrix in regard (left).

The central diagonal on Figs 7–9 shows that contact patterns were highly assortative with age (i.e. participants tend to mix with people of similar age). The 2 secondary parallel diagonals for people with age differences of about 30 years exhibited a high contact rate: children mixing with adults aged 30–39 years and adults mixing with older contacts (>60 years). These diagonal bands were found only in the home matrix (Fig 9), and mostly represent contacts between (grand)parents and their (grand)children. These mixing patterns were maintained for physical contacts and over the different periods.

Men reported significantly fewer contacts with children than women, whatever the contact gender. Men reported significantly fewer contacts with women, whatever the participant or contact age. And boys reported significantly fewer contacts with girls than with boys (Table 2).

**Table 2:** Gender of participant & Contact, without SPC (Supplementary Professional Contacts): Ratio of contact for male participants compared to female, not taking into account the gender of contact and taking into account the gender of contact.

	**Contact (Male & Female)**		
**Male Participant**	**Age**	**≤ 18 years**	**> 18 years**
	**≤ 18 years**	0.88 [0.73;1.06]	**0.85 [0.75;0.96]**
	**> 18 years**	**0.63 [0.48;0.82]**	0.95 [0.86;1.05]
	**Contact (Male)**		
**Male Participant**	**Age**	**≤ 18 years**	**> 18 years**
	**≤ 18 years**	***1*.*42 [1*.*15 ; 1*.*74]***	0.99 [0.83;1.18]
	**> 18 years**	**0.71 [0.52;0.97]**	***1*.*15 [1*.*01;1*.*31]***
	**Contact (Female)**		
**Male Participant**	**Age**	**≤ 18 years**	**> 18 years**
	**≤ 18 years**	**0.51 [0.42;0.62]**	**0.75 [0.67;0.84]**
	**> 18 years**	**0.55 [0.39;0.78]**	**0.79 [0.71;0.87]**

The impact of school closures on an epidemic was estimated by the relative change in R0 on the weekend compared to a weekday and on holidays compared to a regular day. R0 decreased during weekend and holidays by 28%[10%;44%] and 33%[25%;41%], respectively.

### Where do people mix

**Fig 10:**
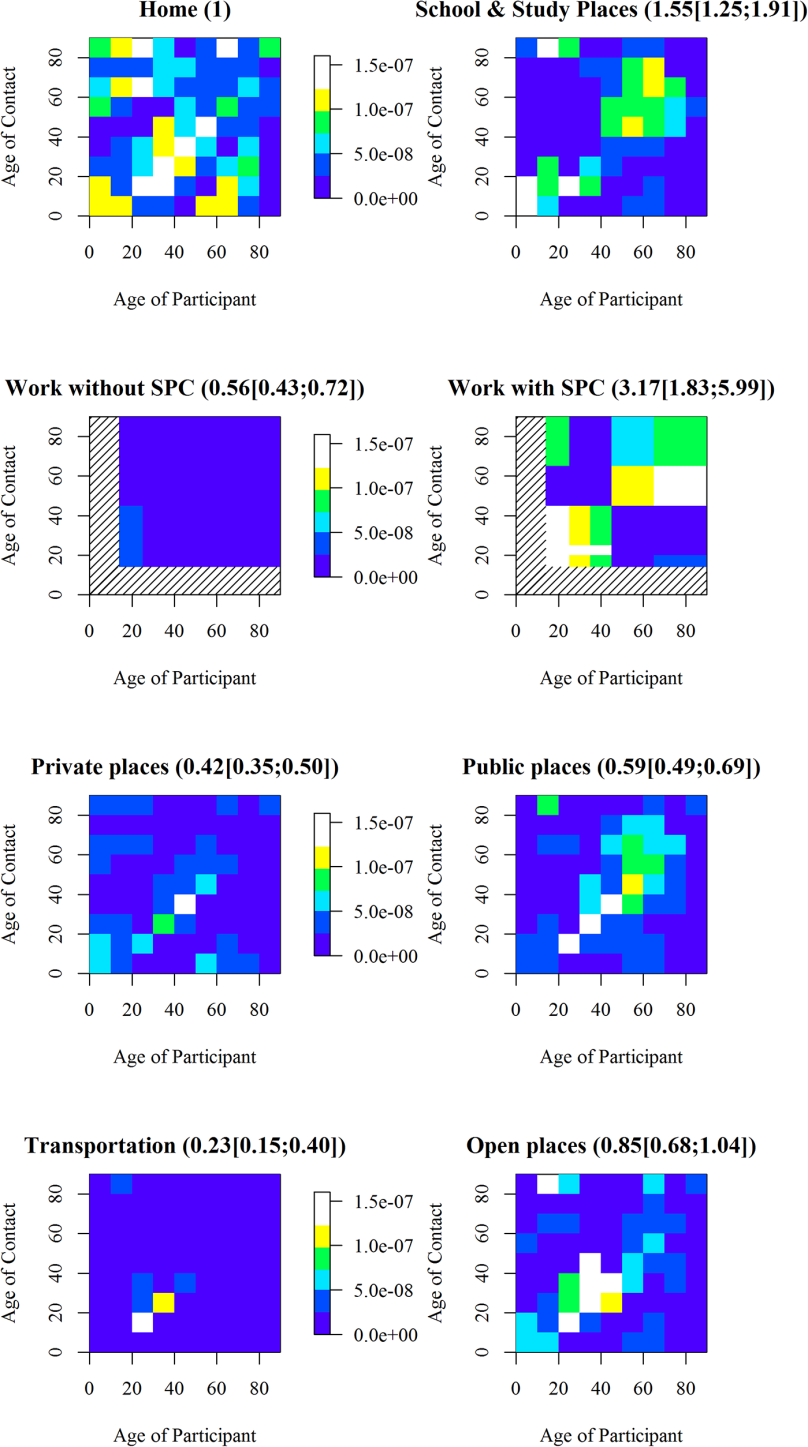
Contact matrices according to location. Numbers are the ratio of contact rate with contact rate at home (95%CI). No smoothing or reciprocity was applied (particular location wouldn’t be the same for a participant and a contact (e.g., at home vs. not at home), the matrices were kept asymmetric).

Contact patterns were different according to location (Fig 10), with most contacts made at school and at home and fewer contacts during transport and in public places. Contact patterns were found to be assortative with age at all locations, and transgenerational mixing occurred mainly at home. With home as a baseline (= 1), the contact ratio according location was higher at school (1.55 [1.25–1.91]), but lower at work (0.56 [0.43–0.72]), in private places (0.42 [0.35–0.50]), in public places (0.59 [0.49–0.69]), in transportation (0.23 [0.15–0.40]), and not different in open spaces (0.85 [0.68–1.04]).

### The professional contacts

The total number of contacts with SPC was 54 378 (9(5–17)) contacts per day), and was 52,042 contacts (9(5–17)) with SPC censored at 134. Hence the reduction of 4% of the total number of contacts involved 22 (1%) participants. Censored participants were similar in gender, age and household size to other participants. SPC increased the variance and attenuated the effect of all variables, except for age <25y, gender, weekend, occupation when employed and the period (Table 1) (S4 Table, S4 Fig). SPC increased the number of contacts during the last period (Design and Actual), and R0 for Actual Period 2 and 3 up to 9%[-27%;111%] and 46%[8%;100%] with full SPC, and to 6%[-26%;84%] and 33%[1%;72%] with censored SPC. With SPC, the contact matrix (Fig 8) showed a wider contact “plateau”, corresponding to less assortative mixing for ages 20–65 years. It resulted in a contact ratio at work higher than in any other location, of 3.17[1.83;5.99](compared to Home as baseline). The mixing pattern specifically at the workplace was mildly assortative by age, but showed a cut-off at 45 years (Fig 10)(individuals under 45 years had contacts mainly with individuals under 45 years, while individuals over 45 years had contacts mainly with individuals over 45 years). The specific number of contact made at work was 3[0;10] and 20[6;38] with SPC. With SPC, R0 decreased during weekends and holidays by 63%[53%;70%] and 20%[-1%;22%], and public transport increased the number of contacts by 96%[28%;198%]).

For participants common to Design Periods 1 and 2, SPC led to similar results except that weekends became significantly associated with fewer contacts.

## Discussion

Comes-F is the first study on temporal variation of social contact patterns to use a contact diary approach. The trend toward more contacts in April-May was significant for design periods but only with SPC for actual periods. The period did not influence the number of contacts among participants common to Design Periods 1 and 2. Hence, actual/design periods in this study showed little influence on the number of contacts compared to age, household size, gender, holidays and weekends. Weather may help to explain these minor differences between the periods. Two recent studies showed that weather conditions could influence the number of contacts and mixing patterns[22,23]. DeStefano suggested similar trends, although without information on statistical significance or mixing patterns[15]. Besides temporal variation, spatial variation such as place of residency, albeit non-significant, could be a confounding effect as weather varies according to both season and primarily latitude, which could influence mixing patterns.

As in POLYMOD, we found that contacts were mostly influenced by age. Contact patterns were likewise highly assortative with age, with a high contact rate for children and adolescents, and a strong child-parent component (Figs 7–9). Mixing with both contacts of similar age and with their parents explains the strong participation of children and adolescents in an epidemic[4] (Figs 8–9). Their high number of contacts favours an important influence of these age groups at the beginning of an epidemic. Additional contacts with other age groups (such as adults aged 35–50 years) lead to a rapid spread among other age groups. Our results present not only similarities with the POLYMOD study, but also with contact survey studies using different methodology, such as an household-structured community cohort [24–26], probability sampling instead of quota [27] or in person (face-to-face) interviews. The highest number of contact was always concentrated on children and teenagers, mixing patterns was assortative by age, and physical contact was more likely to be prolonged, frequent and occurring at home.

Our most original result was the difference in gender, with men having fewer contacts than women and mixing assortative with age and gender. The POLYMOD study[7] found a similar trend, but could not establish its statistical significance. With a different methodology, DeStefano et al[15] found that women had 13% more speaking interactions per day. Nonetheless, to the best of our knowledge, no previous study has ever presented contact matrices according to gender. So far, gender differences in infectious diseases epidemiology have been attributed only to hormonal differences[28] or to differences in risk assessment[29] leading to incomplete reporting[30]. We suggest that the higher participation of women in infectious diseases such as influenza[31] or pertussis[32] could also be attributed to behaviour. Data from Japan[33] showed that several infections were more frequent among males during childhood and among females at an older age. The authors’ hypothesis was that mothers were more likely to stay at home with a sick child and consequently more likely to be exposed to infection. More contact should result in accelerated circulation of pathogens among women. Women should consequently get infected sooner than men, and present a shorter serial interval (time between symptoms onset of a case and its infector). Indeed, in a study on pertussis transmission in Dutch households, the mean serial interval was 20 days when the mother was the infector vs. 28 days when it was the father or a sibling[34]. In a study on household transmission of 2009 influenza in New York City, the secondary attack rate among females was almost twice the rate among males[35]. And in a prospective cohort study, female gender was associated with increased influenza transmission[36]. These differences according to age and gender of both participants and contacts could result from the higher involvement of women in childcare as well as gender differences among professional contacts. In our study gender preference occurred evenly among children, in accordance with a study carried out in a school using wearable sensors[37]. For strong interaction, gender preference increased with grade while for low interaction it decreased for girls and increased for boys. Therefore, different trends on gender preference according to countries in POLYMOD[7] could have led to an average non-significant trend.

In accordance with previous work[3], most contacts occurred at home and school and far fewer in other locations such as transportation. Whatever the location, contacts were assortative with age, but less assortative in places where the contact rate was the highest (home, school or workplace with SPC). The diagonals on the home matrix demonstrated that parent-children contact occurred primarily at home, in accordance to findings from Lapidus[10]. This finding confirms the importance of home and school in the spread of infectious diseases, both because contact rates are high, and because contacts are not limited to a specific age category, thereby allowing the pathogen to spread across age categories. Therefore, home quarantine or school closures would have a higher impact than transport-related measures on the contact rate and the spread of infections.

Most of the studies on school closures have relied on strong assumptions about contact patterns[38–40], or were based on a specific context (multiple non-pharmaceutical measures taken simultaneously, school closures among others), such as 1918 pandemic[41], 2009 H1N1 pandemic in Mexico[42] or SARS in Beijing[43]. Like Hens[21], we used social contact data to quantify the impact of school closure, not relying on data specific to a particular pathogen. Oversampling the number of study days in a holiday period made it possible to study the influence of holidays—also regarded as a proxy for school closures- on mixing patterns. Based on our analysis and in[21], school closure would have more impact on disease transmission in France than in other European countries, as the R0 of an epidemic decreased by 28% and 33% during weekends and holidays, compared to 21% and 17% in the European countries where a significant decrease was found[21]. This difference should be taken into account when estimating the benefits of school closure, which may nonetheless be counterbalanced by a macroeconomic cost that would render such strategies questionable[44].

Unlike Hens et al[19], we found no influence of day-care centre or classroom size on the number of contacts. This could be a methodological issue, resulting from different definitions of the variable, as well as undeniable differences between Belgium and France. Hence, if size of day-care centre or classroom influences the number of contacts, it is neither strong nor linear in our setting.

Inclusion of SPC pronouncedly modified the number of contacts and influencing variables. Partly this effect resulted from our methodology: SPC were included only for weekdays, hence the strong effect of weekends. But it also influenced mixing patterns, as an adult could make contact outside his or her own age category outside the home, notably in the workplace, which could facilitate the spread of a pathogen among different age categories. This observation raises the question of the possible role of the workplace, as well as public transport in pathogen transmission. Gender difference enhanced by SPC could reflect gender difference in rate of employment. In contrast, the influence of SPC on the period is less clear, even though a higher number of contacts increases the power of the analysis and could render a trend significant. One difficulty is that professional contacts-notably when numerous- are unlikely to have the same importance as others regarding infectious disease transmission (e.g. bus driver). Of note, report of professional contacts was limited at 10 in 4 of 8 countries in POLYMOD (20 in Belgium). Issued from a parametric approach, our results are sensitive to extreme values (e.g. participants with a high number of professional contacts). Hence, limiting the maximum number of contacts to 40 a day and modelling the SPC separately may effectively help to limit the effect of these outliers. That said, while limiting the number of reported contacts facilitates completion of the diary, it inevitably leads to artificial boundaries (Fig 2). Therefore, separate modelling of SPC (see Hens et al[19]) may represent an optimal trade-off between relevance and feasibility.

Whereas the impact of professional contacts on social mixing patterns was assessed, more detailed future analyses may require the use of additional poststratification for employment given the moderate undersampling of employed people in the 50–64y category. When more refined analyses with several poststratification dimensions are of interest, care should be taken with highly variable poststratification weights [45](see e.g. Vandendijck et al.).

The reporting of contacts for 2 consecutive days offered a large amount of data resulting in the largest contact survey for a country. But this positive factor is counteracted by fatigue in reporting, with fewer reported contacts during the second day. Smieszek et al[46] showed higher underreporting for highly connected individuals than for isolated individuals. As the proportion of short and non-intense contacts increased with the total number of contact partners, underreporting of contacts was correlated with contact duration. The fact that participants were slightly older than non-participants could be related to their being less active and/or employed, with less time to participate. Therefore, there is a limit to the amount of information we could request in view of achieving optimal accuracy.

We have presented the first large population contact survey in France, and one of the largest contact surveys of its kind. It improves our understanding on the spread of infectious diseases, on the role of some age categories and on the impact of school closures in France. It raises more fundamental questions on the optimal design of those surveys, on the role of professional contacts and locations, and on the gender difference in the epidemiology of infectious diseases. Finally, it provides some basic material to be used in applied model-based analyses.

## Supporting Information

S1 Fig Children and adult diaries. (PDF)

S2 Fig Influencing variables graph. (TIF)

S3 Fig Degree distribution graph frequency-based. (PDF)

S4 Fig Contact description with SPC (Supplementary Professional Contacts). (TIF)

S1 Text Next Generation Matrix and R0 calculation. (DOCX)

S2 Text Supplementary Professional contact modelling methodology. (DOCX)

S3 Text Design issues. (DOCX)

S1 Table Factors influencing the number of contacts, with censoring at 29 similarly to Mossong et al 2008 (non-linear model). (DOCX)

S2 Table Employment and school enrolment rates. (DOCX)

S3 Table Base-case contact matrix with age categories for all contact and for skin contact only. (DOCX)

S4 Table Gender table with SPC (Supplementary Professional Contacts). (DOCX)
