# Supplementary material for: The French Connection: The First Large Population-Based Contact Survey in France Relevant for the Spread of Infectious Diseases
Source: PLoS One. 2015 Jul 15;10(7):e0133203. doi: 10.1371/journal.pone.0133203 (PMC4503306; doi:10.1371/journal.pone.0133203)
Supplement: S1 Fig — (PDF) [file pone.0133203.s001.pdf]

UNIVERSITÉ CATHOLIQUE DE LILLE 1875

V2

Ipsos

A

ÉTUDE CONTACTS - CoMEs-F

Quelques données personnelles sur vous-même

1. Indiquez le code postal de votre résidence principale :

2. Vous êtes… :

- une femme.................☐1
- un homme..................☐2

3. Indiquez votre âge et l'âge des autres personnes **résidant dans votre logement** : en commençant par vous puis en poursuivant par la personne la plus âgée.

Faites figurer dans ce tableau toutes les personnes résidant dans votre logement.

	Âge		Âge
**Vous-même**	ans	6ème personne	ans
2ème personne	ans	7ème personne	ans
3ème personne	ans	8ème personne	ans
4ème personne	ans	9ème personne	ans
5ème personne	ans	10ème personne	ans

4. Quel est le diplôme le plus élevé que vous ayez obtenu ?
(une seule réponse possible)

- Aucun diplôme / Certificat d'étude primaires ....☐1
- BEPC, brevet ....☐2
- CAP, brevet de compagnon, BEP ....☐3
- Baccalauréat (général, technique ou professionnel)......................☐4
- BTS et diplôme de l'enseignement supérieur du 1er cycle (jusqu'au BAC+3)................................☐5
- Diplôme de l'enseignement supérieur du 2ème ou du 3ème cycle, diplômés des grandes écoles….........☐6

5. Indiquez vos **modes de déplacements** privilégiés
Vous pouvez cocher plusieurs réponses pour la semaine et plusieurs réponses pour le week-end / vacances

	La semaine	Le week-end et en vacances
Voiture particulière ou deux roues	☐1	☐1
Transport collectif (bus, métro, train…)	☐2	☐2
À pied	☐3	☐3

6. Quelle est votre **situation professionnelle actuelle** ?
(une seule réponse possible)

- Agriculteur ....☐01
- Artisan, commerçant, chef d'entreprise.......☐02
- Cadre, profession intellectuelle supérieure....☐03
- Profession intermédiaire.............................☐04
- Employé ....☐05
- Ouvrier...☐06

Passez à **7.**

- Élève ou étudiant ....☐07 → *Passez à* **9.**

- Retraité...☐08
- À la recherche d'un emploi.........................☐09
- Autre sans activité (personne au foyer…)....☐10

Passez à la page **3.**

➛ Vous exercez actuellement une profession :

(Si vous exercez actuellement une profession)

7. Dans quel **secteur d'activité** travaillez-vous ?
(une seule réponse possible)

- Agriculture, sylviculture, pêche .... ☐ 01
- Industrie agricole et alimentaire .... ☐ 02
- Autre industrie .... ☐ 03
- Énergie .... ☐ 04
- Construction .... ☐ 05
- Commerce .... ☐ 06
- Activités financières et immobilières .... ☐ 07
- Services aux entreprises .... ☐ 08
- Services aux personnes .... ☐ 09
- Éducation, santé, action social.................... ☐ 10
- Administration .... ☐ 11

8. Exercez-vous une profession qui entraîne beaucoup de contacts *(clients pour les commerciaux, les coiffeurs…, patients pour les personnels soignants, élèves ou étudiants pour les professeurs…)* ?

- Oui .... ☐ 1 ➛ *Passez à* **8a** et **suivantes**
- Non .... ☐ 2 ➛ *Allez directement en page 3*

8a. À combien estimez-vous en moyenne le nombre de ces personnes (clients, patients, élèves,...) que vous rencontrez par jour :

[ ] personnes

8b. Ces contacts professionnels se situent plutôt dans les groupes suivants :
(plusieurs réponses possibles)

- 0-3 ans .... ☐ 1
- 3-10 ans .... ☐ 2
- 11-17 ans .... ☐ 3
- 18-64 ans .... ☐ 4
- plus de 64 ans .... ☐ 5

8c. Si vous estimez le nombre de ces contacts à plus de 20, nous vous prions de ne pas énumérer vos contacts professionnels dans votre journal, et de seulement indiquer les autres contacts.

- J'ai plus de 20 contacts professionnels .... ☐ 1
- J'ai moins de 20 contacts professionnels .... ☐ 2

➛ Vous êtes élève ou étudiant :

9. Combien y a-t-il d'élèves / étudiants dans votre classe ?
(une seule réponse possible)

- il y a moins de 20 élèves/étudiants dans la classe... ☐ 1
- entre 20 et 30 élèves/étudiants dans la classe......... ☐ 2
- plus de 30 élèves/étudiants dans la classe .... ☐ 3

10. Lorsque vous êtes en cours, vous …?
(une seule réponse possible)

- ne mangez jamais à la cantine / au restaurant universitaire.. ☐ 1
- mangez occasionnellement à la cantine / au restaurant universitaire.. ☐ 2
- êtes demi-pensionnaire .... ☐ 3
- êtes interne .... ☐ 4

Guide de remplissage du carnet

Nous vous prions d'indiquer dans ce journal toutes les personnes avec lesquelles vous avez été en **contact direct** et que vous avez rencontrées durant **les deux journées retenues**.

① **<u>L'enquête concerne les contacts directs</u>**

Qu'est ce qu'un contact direct ?

- Un contact veut dire que vous avez **parlé avec quelqu'un en sa présence physique et à une distance inférieure de 2 mètres.**
  - Les contacts par téléphone ou internet sont exclus,
  - les contacts ayant donné à une discussion non rapprochée (plus de 2 mètres) ne doivent pas être pris en compte.
- Un contact peut aussi être physique : **toucher la peau** de l'autre personne (se donner ou se serrer la main, s'embrasser, se donner l'accolade…).

On ne retient pas les contacts avec des animaux.

② **<u>Une ligne par personne contactée</u>**

Il faut utiliser une seule ligne par personne contactée.

Si vous avez rencontré la même personne plusieurs fois dans la même journée, ne remplissez qu'une seule ligne en estimant au total combien de temps vous avez passé avec cette personne dans la journée, comme cela est donné en exemple.

<u>Exemple 1</u> : Vous avez parlé 10 minutes à votre fils de 9 ans en le conduisant à l'école le matin. Le soir, vous l'avez accompagné pendant ses devoirs, vous avez joué ensemble et discuté ensemble pendant le repas entre 18 et 20 heures (pendant 2 heures) et vous l'avez embrassé avant d'aller au lit.

③ **<u>Conseils généraux sur le remplissage du questionnaire</u>**

La réponse au questionnaire sera plus facile si vous prenez des notes au fur et à mesure au cours de la journée (toutes les 2 heures ou après les repas par exemple). Vous pouvez aussi vous appuyer sur **votre agenda**.

Vous pouvez décrire vos contacts directs **par ordre chronologique**, en commençant par la personne que vous avez rencontrée en premier lors de la journée et en continuant avec toutes les autres personnes dont vous vous souvenez en fonction des activités de la journée.

④ **<u>J'ai plus de 20 contacts professionnels dans la journée</u>**

J'ai une profession qui m'amène à rencontrer de nombreuses personnes. Dois-je limiter le nombre de contacts décrits ?

Si vous avez estimé le nombre de ces contacts professionnels à plus de 20, nous vous prions de ne pas énumérer vos contacts professionnels dans votre journal, et de seulement indiquer les autres contacts.
Si vous êtes dans cette situation, n'oubliez pas de le préciser dans le questionnaire dans le point 12c.

⑤ **<u>Les difficultés possibles</u>**

Je ne connais pas l'âge de la personne avec qui j'ai eu un contact direct ?

Donnez **une estimation de l'âge de la personne** avec qui vous avez eu un contact

<u>Exemple 2</u> : Vous avez parlé à une vendeuse âgée d'une quarantaine d'années dans votre magasin de chaussures préféré, où vous allez plusieurs fois par an.

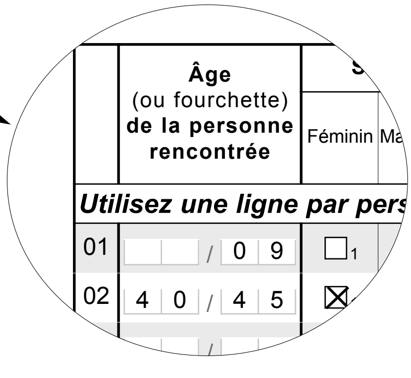

Que dois-je faire si j'ai eu plusieurs contacts directs avec la même personne pendant la journée ?

Utilisez une seule ligne et estimez le temps total que vous avez passé ensemble lors de la journée attribuée.

Dans l'exemple 1 : Vous avez parlé 10 minutes à votre fils le matin. Le soir, entre le temps passé aux devoirs, à jouer et discuter et à manger, vous avez passé plus de 2 heures 'en contact' avec lui ➔ ***la durée des contacts (avec votre fils) à indiquer pour cette journée sera '1-4h'***

⑥ **<u>Une fois le questionnaire rempli</u>**

Quand vous avez décrit votre dernier contact direct, nous vous conseillons de réfléchir encore une fois afin de vérifier que vous n'avez pas oublié une activité où un contact. Votre agenda pourra être utile à cet effet.

Au moment de remplir les grilles, n'hésitez pas à vous référez au document 'Aide au remplissage' - tous les exemples sont illustrés et les consignes rappelées - nous espérons que cela vous aidera.

Jour 1 : ___ ___

Carnet des contacts

	Âge (ou fourchette) **de la personne rencontrée**	**Sexe**		**Lieu(x) de contacts** noter <u>tous les lieux</u> où la personne a été en contact avec vous							**À quelle fréquence rencontrez-vous cette personne ?**					**A-t-elle touché votre peau ?**		**Durée totale des contacts avec une même personne**				
		Féminin	Masculin	Domicile, véhicule ou autres lieux privatifs	École, collège, lycée ou tout autre lieu d'études	Lieux de travail clos (bureau, atelier)	Chez des proches en lieux clos	Autre lieux clos (restaurant, commerce, …)	Transport collectif	Lieux ouverts (parc, rue) y compris pour le travail (chantier, voie publique…)	(presque) chaque jour	Quelques fois par semaine	Quelques fois par mois	Quelques fois par an ou moins souvent	1ère fois	Oui	Non	Moins de 5 min	5 -15 min	15 min - 1 h	1-4 h	4H ou plus
Utilisez une ligne par personne rencontrée et avec laquelle vous avez eu au moins un 'contact'																						
01	/	☐1	☐2	☐1	☐2	☐3	☐4	☐5	☐6	☐7	☐1	☐2	☐3	☐4	☐5	☐1	☐2	☐1	☐2	☐3	☐4	☐5
02	/	☐1	☐2	☐1	☐2	☐3	☐4	☐5	☐6	☐7	☐1	☐2	☐3	☐4	☐5	☐1	☐2	☐1	☐2	☐3	☐4	☐5
03	/	☐1	☐2	☐1	☐2	☐3	☐4	☐5	☐6	☐7	☐1	☐2	☐3	☐4	☐5	☐1	☐2	☐1	☐2	☐3	☐4	☐5
04	/	☐1	☐2	☐1	☐2	☐3	☐4	☐5	☐6	☐7	☐1	☐2	☐3	☐4	☐5	☐1	☐2	☐1	☐2	☐3	☐4	☐5
05	/	☐1	☐2	☐1	☐2	☐3	☐4	☐5	☐6	☐7	☐1	☐2	☐3	☐4	☐5	☐1	☐2	☐1	☐2	☐3	☐4	☐5
06	/	☐1	☐2	☐1	☐2	☐3	☐4	☐5	☐6	☐7	☐1	☐2	☐3	☐4	☐5	☐1	☐2	☐1	☐2	☐3	☐4	☐5
07	/	☐1	☐2	☐1	☐2	☐3	☐4	☐5	☐6	☐7	☐1	☐2	☐3	☐4	☐5	☐1	☐2	☐1	☐2	☐3	☐4	☐5
08	/	☐1	☐2	☐1	☐2	☐3	☐4	☐5	☐6	☐7	☐1	☐2	☐3	☐4	☐5	☐1	☐2	☐1	☐2	☐3	☐4	☐5
09	/	☐1	☐2	☐1	☐2	☐3	☐4	☐5	☐6	☐7	☐1	☐2	☐3	☐4	☐5	☐1	☐2	☐1	☐2	☐3	☐4	☐5
10	/	☐1	☐2	☐1	☐2	☐3	☐4	☐5	☐6	☐7	☐1	☐2	☐3	☐4	☐5	☐1	☐2	☐1	☐2	☐3	☐4	☐5
11	/	☐1	☐2	☐1	☐2	☐3	☐4	☐5	☐6	☐7	☐1	☐2	☐3	☐4	☐5	☐1	☐2	☐1	☐2	☐3	☐4	☐5
12	/	☐1	☐2	☐1	☐2	☐3	☐4	☐5	☐6	☐7	☐1	☐2	☐3	☐4	☐5	☐1	☐2	☐1	☐2	☐3	☐4	☐5
13	/	☐1	☐2	☐1	☐2	☐3	☐4	☐5	☐6	☐7	☐1	☐2	☐3	☐4	☐5	☐1	☐2	☐1	☐2	☐3	☐4	☐5
14	/	☐1	☐2	☐1	☐2	☐3	☐4	☐5	☐6	☐7	☐1	☐2	☐3	☐4	☐5	☐1	☐2	☐1	☐2	☐3	☐4	☐5
15	/	☐1	☐2	☐1	☐2	☐3	☐4	☐5	☐6	☐7	☐1	☐2	☐3	☐4	☐5	☐1	☐2	☐1	☐2	☐3	☐4	☐5
16	/	☐1	☐2	☐1	☐2	☐3	☐4	☐5	☐6	☐7	☐1	☐2	☐3	☐4	☐5	☐1	☐2	☐1	☐2	☐3	☐4	☐5
17	/	☐1	☐2	☐1	☐2	☐3	☐4	☐5	☐6	☐7	☐1	☐2	☐3	☐4	☐5	☐1	☐2	☐1	☐2	☐3	☐4	☐5
18	/	☐1	☐2	☐1	☐2	☐3	☐4	☐5	☐6	☐7	☐1	☐2	☐3	☐4	☐5	☐1	☐2	☐1	☐2	☐3	☐4	☐5
19	/	☐1	☐2	☐1	☐2	☐3	☐4	☐5	☐6	☐7	☐1	☐2	☐3	☐4	☐5	☐1	☐2	☐1	☐2	☐3	☐4	☐5
20	/	☐1	☐2	☐1	☐2	☐3	☐4	☐5	☐6	☐7	☐1	☐2	☐3	☐4	☐5	☐1	☐2	☐1	☐2	☐3	☐4	☐5

Jour 1 *(suite…)*

	Âge (ou fourchette) **de la personne rencontrée**	**Sexe**		**Lieu(x) de contacts** noter <u>tous les lieux</u> où la personne a été en contact avec vous							**À quelle fréquence rencontrez-vous cette personne ?**					**A-t-elle touché votre peau ?**		**Durée totale des contacts avec une même personne**				
		Féminin	Masculin	Domicile, véhicule ou autres lieux privatifs	École, collège, lycée ou tout autre lieu d'études	Lieux de travail clos (bureau, atelier)	Chez des proches en lieux clos	Autre lieux clos (restaurant, commerce, …)	Transport collectif	Lieux ouverts (parc, rue) y compris pour le travail (chantier, voie publique…)	(presque) chaque jour	Quelques fois par semaine	Quelques fois par mois	Quelques fois par an ou moins souvent	1ère fois	Oui	Non	Moins de 5 min	5 -15 min	15 min - 1 h	1-4 h	4H ou plus
21	__ __ / __ __	☐1	☐2	☐1	☐2	☐3	☐4	☐5	☐6	☐7	☐1	☐2	☐3	☐4	☐5	☐1	☐2	☐1	☐2	☐3	☐4	☐5
22	__ __ / __ __	☐1	☐2	☐1	☐2	☐3	☐4	☐5	☐6	☐7	☐1	☐2	☐3	☐4	☐5	☐1	☐2	☐1	☐2	☐3	☐4	☐5
23	__ __ / __ __	☐1	☐2	☐1	☐2	☐3	☐4	☐5	☐6	☐7	☐1	☐2	☐3	☐4	☐5	☐1	☐2	☐1	☐2	☐3	☐4	☐5
24	__ __ / __ __	☐1	☐2	☐1	☐2	☐3	☐4	☐5	☐6	☐7	☐1	☐2	☐3	☐4	☐5	☐1	☐2	☐1	☐2	☐3	☐4	☐5
25	__ __ / __ __	☐1	☐2	☐1	☐2	☐3	☐4	☐5	☐6	☐7	☐1	☐2	☐3	☐4	☐5	☐1	☐2	☐1	☐2	☐3	☐4	☐5
26	__ __ / __ __	☐1	☐2	☐1	☐2	☐3	☐4	☐5	☐6	☐7	☐1	☐2	☐3	☐4	☐5	☐1	☐2	☐1	☐2	☐3	☐4	☐5
27	__ __ / __ __	☐1	☐2	☐1	☐2	☐3	☐4	☐5	☐6	☐7	☐1	☐2	☐3	☐4	☐5	☐1	☐2	☐1	☐2	☐3	☐4	☐5
28	__ __ / __ __	☐1	☐2	☐1	☐2	☐3	☐4	☐5	☐6	☐7	☐1	☐2	☐3	☐4	☐5	☐1	☐2	☐1	☐2	☐3	☐4	☐5
29	__ __ / __ __	☐1	☐2	☐1	☐2	☐3	☐4	☐5	☐6	☐7	☐1	☐2	☐3	☐4	☐5	☐1	☐2	☐1	☐2	☐3	☐4	☐5
30	__ __ / __ __	☐1	☐2	☐1	☐2	☐3	☐4	☐5	☐6	☐7	☐1	☐2	☐3	☐4	☐5	☐1	☐2	☐1	☐2	☐3	☐4	☐5
31	__ __ / __ __	☐1	☐2	☐1	☐2	☐3	☐4	☐5	☐6	☐7	☐1	☐2	☐3	☐4	☐5	☐1	☐2	☐1	☐2	☐3	☐4	☐5
32	__ __ / __ __	☐1	☐2	☐1	☐2	☐3	☐4	☐5	☐6	☐7	☐1	☐2	☐3	☐4	☐5	☐1	☐2	☐1	☐2	☐3	☐4	☐5
33	__ __ / __ __	☐1	☐2	☐1	☐2	☐3	☐4	☐5	☐6	☐7	☐1	☐2	☐3	☐4	☐5	☐1	☐2	☐1	☐2	☐3	☐4	☐5
34	__ __ / __ __	☐1	☐2	☐1	☐2	☐3	☐4	☐5	☐6	☐7	☐1	☐2	☐3	☐4	☐5	☐1	☐2	☐1	☐2	☐3	☐4	☐5
35	__ __ / __ __	☐1	☐2	☐1	☐2	☐3	☐4	☐5	☐6	☐7	☐1	☐2	☐3	☐4	☐5	☐1	☐2	☐1	☐2	☐3	☐4	☐5
36	__ __ / __ __	☐1	☐2	☐1	☐2	☐3	☐4	☐5	☐6	☐7	☐1	☐2	☐3	☐4	☐5	☐1	☐2	☐1	☐2	☐3	☐4	☐5
37	__ __ / __ __	☐1	☐2	☐1	☐2	☐3	☐4	☐5	☐6	☐7	☐1	☐2	☐3	☐4	☐5	☐1	☐2	☐1	☐2	☐3	☐4	☐5
38	__ __ / __ __	☐1	☐2	☐1	☐2	☐3	☐4	☐5	☐6	☐7	☐1	☐2	☐3	☐4	☐5	☐1	☐2	☐1	☐2	☐3	☐4	☐5
39	__ __ / __ __	☐1	☐2	☐1	☐2	☐3	☐4	☐5	☐6	☐7	☐1	☐2	☐3	☐4	☐5	☐1	☐2	☐1	☐2	☐3	☐4	☐5
40	__ __ / __ __	☐1	☐2	☐1	☐2	☐3	☐4	☐5	☐6	☐7	☐1	☐2	☐3	☐4	☐5	☐1	☐2	☐1	☐2	☐3	☐4	☐5

Jour 2 : ☐☐ ☐☐

Carnet des contacts

	Âge (ou fourchette) de la personne rencontrée	Sexe		Lieu(x) de contacts noter tous les lieux où la personne a été en contact avec vous							À quelle fréquence rencontrez-vous cette personne ?					A-t-elle touché votre peau ?		Durée totale des contacts avec une même personne				
		Féminin	Masculin	Domicile, véhicule ou autres lieux privatifs	École, collège, lycée ou tout autre lieu d'études	Lieux de travail clos (bureau, atelier)	Chez des proches en lieux clos	Autre lieux clos (restaurant, commerce, …)	Transport collectif	Lieux ouverts (parc, rue) y compris pour le travail (chantier, voie publique…)	(presque) chaque jour	Quelques fois par semaine	Quelques fois par mois	Quelques fois par an ou moins souvent	1ère fois	Oui	Non	Moins de 5 min	5 -15 min	15 min - 1 h	1-4 h	4H ou plus
Utilisez une ligne par personne rencontrée et avec laquelle vous avez eu au moins un 'contact'																						
**01**	/	☐1	☐2	☐1	☐2	☐3	☐4	☐5	☐6	☐7	☐1	☐2	☐3	☐4	☐5	☐1	☐2	☐1	☐2	☐3	☐4	☐5
**02**	/	☐1	☐2	☐1	☐2	☐3	☐4	☐5	☐6	☐7	☐1	☐2	☐3	☐4	☐5	☐1	☐2	☐1	☐2	☐3	☐4	☐5
**03**	/	☐1	☐2	☐1	☐2	☐3	☐4	☐5	☐6	☐7	☐1	☐2	☐3	☐4	☐5	☐1	☐2	☐1	☐2	☐3	☐4	☐5
**04**	/	☐1	☐2	☐1	☐2	☐3	☐4	☐5	☐6	☐7	☐1	☐2	☐3	☐4	☐5	☐1	☐2	☐1	☐2	☐3	☐4	☐5
**05**	/	☐1	☐2	☐1	☐2	☐3	☐4	☐5	☐6	☐7	☐1	☐2	☐3	☐4	☐5	☐1	☐2	☐1	☐2	☐3	☐4	☐5
**06**	/	☐1	☐2	☐1	☐2	☐3	☐4	☐5	☐6	☐7	☐1	☐2	☐3	☐4	☐5	☐1	☐2	☐1	☐2	☐3	☐4	☐5
**07**	/	☐1	☐2	☐1	☐2	☐3	☐4	☐5	☐6	☐7	☐1	☐2	☐3	☐4	☐5	☐1	☐2	☐1	☐2	☐3	☐4	☐5
**08**	/	☐1	☐2	☐1	☐2	☐3	☐4	☐5	☐6	☐7	☐1	☐2	☐3	☐4	☐5	☐1	☐2	☐1	☐2	☐3	☐4	☐5
**09**	/	☐1	☐2	☐1	☐2	☐3	☐4	☐5	☐6	☐7	☐1	☐2	☐3	☐4	☐5	☐1	☐2	☐1	☐2	☐3	☐4	☐5
**10**	/	☐1	☐2	☐1	☐2	☐3	☐4	☐5	☐6	☐7	☐1	☐2	☐3	☐4	☐5	☐1	☐2	☐1	☐2	☐3	☐4	☐5
**11**	/	☐1	☐2	☐1	☐2	☐3	☐4	☐5	☐6	☐7	☐1	☐2	☐3	☐4	☐5	☐1	☐2	☐1	☐2	☐3	☐4	☐5
**12**	/	☐1	☐2	☐1	☐2	☐3	☐4	☐5	☐6	☐7	☐1	☐2	☐3	☐4	☐5	☐1	☐2	☐1	☐2	☐3	☐4	☐5
**13**	/	☐1	☐2	☐1	☐2	☐3	☐4	☐5	☐6	☐7	☐1	☐2	☐3	☐4	☐5	☐1	☐2	☐1	☐2	☐3	☐4	☐5
**14**	/	☐1	☐2	☐1	☐2	☐3	☐4	☐5	☐6	☐7	☐1	☐2	☐3	☐4	☐5	☐1	☐2	☐1	☐2	☐3	☐4	☐5
**15**	/	☐1	☐2	☐1	☐2	☐3	☐4	☐5	☐6	☐7	☐1	☐2	☐3	☐4	☐5	☐1	☐2	☐1	☐2	☐3	☐4	☐5
**16**	/	☐1	☐2	☐1	☐2	☐3	☐4	☐5	☐6	☐7	☐1	☐2	☐3	☐4	☐5	☐1	☐2	☐1	☐2	☐3	☐4	☐5
**17**	/	☐1	☐2	☐1	☐2	☐3	☐4	☐5	☐6	☐7	☐1	☐2	☐3	☐4	☐5	☐1	☐2	☐1	☐2	☐3	☐4	☐5
**18**	/	☐1	☐2	☐1	☐2	☐3	☐4	☐5	☐6	☐7	☐1	☐2	☐3	☐4	☐5	☐1	☐2	☐1	☐2	☐3	☐4	☐5
**19**	/	☐1	☐2	☐1	☐2	☐3	☐4	☐5	☐6	☐7	☐1	☐2	☐3	☐4	☐5	☐1	☐2	☐1	☐2	☐3	☐4	☐5
**20**	/	☐1	☐2	☐1	☐2	☐3	☐4	☐5	☐6	☐7	☐1	☐2	☐3	☐4	☐5	☐1	☐2	☐1	☐2	☐3	☐4	☐5

Jour 2 *(suite…)*

	Âge (ou fourchette) **de la personne rencontrée**	Sexe		Lieu(x) de contacts<br>noter tous les lieux où la personne a été en contact avec vous							À quelle fréquence rencontrez-vous cette personne ?					A-t-elle touché votre peau ?		Durée totale des contacts avec une même personne				
		Féminin	Masculin	Domicile, véhicule ou autres lieux privatifs	École, collège, lycée ou tout autre lieu d'études	Lieux de travail clos (bureau, atelier)	Chez des proches en lieux clos	Autre lieux clos (restaurant, commerce, …)	Transport collectif	Lieux ouverts (parc, rue) y compris pour le travail (chantier, voie publique…)	(presque) chaque jour	Quelques fois par semaine	Quelques fois par mois	Quelques fois par an ou moins souvent	1ère fois	Oui	Non	Moins de 5 min	5 -15 min	15 min - 1 h	1-4 h	4H ou plus
**21**	__ / __	☐1	☐2	☐1	☐2	☐3	☐4	☐5	☐6	☐7	☐1	☐2	☐3	☐4	☐5	☐1	☐2	☐1	☐2	☐3	☐4	☐5
**22**	__ / __	☐1	☐2	☐1	☐2	☐3	☐4	☐5	☐6	☐7	☐1	☐2	☐3	☐4	☐5	☐1	☐2	☐1	☐2	☐3	☐4	☐5
**23**	__ / __	☐1	☐2	☐1	☐2	☐3	☐4	☐5	☐6	☐7	☐1	☐2	☐3	☐4	☐5	☐1	☐2	☐1	☐2	☐3	☐4	☐5
**24**	__ / __	☐1	☐2	☐1	☐2	☐3	☐4	☐5	☐6	☐7	☐1	☐2	☐3	☐4	☐5	☐1	☐2	☐1	☐2	☐3	☐4	☐5
**25**	__ / __	☐1	☐2	☐1	☐2	☐3	☐4	☐5	☐6	☐7	☐1	☐2	☐3	☐4	☐5	☐1	☐2	☐1	☐2	☐3	☐4	☐5
**26**	__ / __	☐1	☐2	☐1	☐2	☐3	☐4	☐5	☐6	☐7	☐1	☐2	☐3	☐4	☐5	☐1	☐2	☐1	☐2	☐3	☐4	☐5
**27**	__ / __	☐1	☐2	☐1	☐2	☐3	☐4	☐5	☐6	☐7	☐1	☐2	☐3	☐4	☐5	☐1	☐2	☐1	☐2	☐3	☐4	☐5
**28**	__ / __	☐1	☐2	☐1	☐2	☐3	☐4	☐5	☐6	☐7	☐1	☐2	☐3	☐4	☐5	☐1	☐2	☐1	☐2	☐3	☐4	☐5
**29**	__ / __	☐1	☐2	☐1	☐2	☐3	☐4	☐5	☐6	☐7	☐1	☐2	☐3	☐4	☐5	☐1	☐2	☐1	☐2	☐3	☐4	☐5
**30**	__ / __	☐1	☐2	☐1	☐2	☐3	☐4	☐5	☐6	☐7	☐1	☐2	☐3	☐4	☐5	☐1	☐2	☐1	☐2	☐3	☐4	☐5
**31**	__ / __	☐1	☐2	☐1	☐2	☐3	☐4	☐5	☐6	☐7	☐1	☐2	☐3	☐4	☐5	☐1	☐2	☐1	☐2	☐3	☐4	☐5
**32**	__ / __	☐1	☐2	☐1	☐2	☐3	☐4	☐5	☐6	☐7	☐1	☐2	☐3	☐4	☐5	☐1	☐2	☐1	☐2	☐3	☐4	☐5
**33**	__ / __	☐1	☐2	☐1	☐2	☐3	☐4	☐5	☐6	☐7	☐1	☐2	☐3	☐4	☐5	☐1	☐2	☐1	☐2	☐3	☐4	☐5
**34**	__ / __	☐1	☐2	☐1	☐2	☐3	☐4	☐5	☐6	☐7	☐1	☐2	☐3	☐4	☐5	☐1	☐2	☐1	☐2	☐3	☐4	☐5
**35**	__ / __	☐1	☐2	☐1	☐2	☐3	☐4	☐5	☐6	☐7	☐1	☐2	☐3	☐4	☐5	☐1	☐2	☐1	☐2	☐3	☐4	☐5
**36**	__ / __	☐1	☐2	☐1	☐2	☐3	☐4	☐5	☐6	☐7	☐1	☐2	☐3	☐4	☐5	☐1	☐2	☐1	☐2	☐3	☐4	☐5
**37**	__ / __	☐1	☐2	☐1	☐2	☐3	☐4	☐5	☐6	☐7	☐1	☐2	☐3	☐4	☐5	☐1	☐2	☐1	☐2	☐3	☐4	☐5
**38**	__ / __	☐1	☐2	☐1	☐2	☐3	☐4	☐5	☐6	☐7	☐1	☐2	☐3	☐4	☐5	☐1	☐2	☐1	☐2	☐3	☐4	☐5
**39**	__ / __	☐1	☐2	☐1	☐2	☐3	☐4	☐5	☐6	☐7	☐1	☐2	☐3	☐4	☐5	☐1	☐2	☐1	☐2	☐3	☐4	☐5
**40**	__ / __	☐1	☐2	☐1	☐2	☐3	☐4	☐5	☐6	☐7	☐1	☐2	☐3	☐4	☐5	☐1	☐2	☐1	☐2	☐3	☐4	☐5

Notes

Un événement particulier s'est déroulé pendant les jours d'enquête, vous vous êtes posé des questions lors du remplissage, vous souhaitez nous apporter des précisions… merci d'écrire vos commentaires ci-dessous…

Pour vous aider

N'hésitez pas à relire **les explications qui se trouvent en page 3 de votre carnet**, ou à vous reporter **aux exemples qui figurent sur la feuille 'Aide au remplissage'**.

Un N° de téléphone est à votre disposition : vous pouvez nous contacter aux heures de bureau si vous avez des questions ou des informations à nous communiquer.

Pour de plus amples informations, vous pouvez aussi nous téléphoner ou nous écrire.

Email : **enquete.contacts@ipsos.com**

Tél : **08 00 97 07 32**

MERCI

d'avoir participé à cette enquête, aux noms d'Ipsos et
de l'équipe de recherche en charge de ce projet.

UNIVERSITÉ CATHOLIQUE DE LILLE 1875

E |_|_|_|_|_|_|_|_|_|_| V2

Ipsos

ÉTUDE CONTACTS - CoMEs-F

Quelques données personnelles sur l'enfant qui participe à l'enquête

1. Indiquez son âge : |_|_| ans

2. Indiquez son sexe :

- Fille..☐1
- Garçon ....☐2

3. Quel est votre lien avec l'enfant ?
(une seule réponse possible)

- Mère.......................................☐1
- Père.......................................☐2
- Ni son père, ni sa mère, mais son responsable légal ...☐3

4. Quand votre enfant se déplace, quels sont ses **modes de déplacement** privilégiés (avec ou sans vous) ?
Vous pouvez cocher plusieurs réponses pour la semaine et plusieurs réponses pour le week-end / vacances

	La semaine	Le week-end et en vacances
Voiture particulière ou deux roues	☐1	☐1
Transport collectif (bus, métro, train…)	☐2	☐2
À pied	☐3	☐3

Quelques données personnelles sur vous-même

5. Indiquez le code postal de votre résidence principale :

|_|_|_|_|_|

6. Vous êtes :

- Une femme ....☐1
- Un homme ....☐2

7. Indiquez l'âge de **toutes les personnes résidant dans votre foyer** :
- En commençant par vous
- Puis par l'enfant sélectionné pour l'enquête
- Et en poursuivant par les autres personnes : de la plus âgée à la plus jeune.

	Âge		Âge
**Vous-même**	|_|_| ans	6ème personne	|_|_| ans
**Enfant sélectionné**	|_|_| ans	7ème personne	|_|_| ans
3ème personne	|_|_| ans	8ème personne	|_|_| ans
4ème personne	|_|_| ans	9ème personne	|_|_| ans
5ème personne	|_|_| ans	10ème personne	|_|_| ans

8. Quel est le diplôme le plus élevé que vous ayez obtenu ?
(une seule réponse possible)

- Aucun diplôme / Certificat d'étude primaires ....☐1
- BEPC, brevet ....☐2
- CAP, brevet de compagnon, BEP ....☐3
- Baccalauréat (général, technique ou professionnel)......................☐4
- BTS et diplôme de l'enseignement supérieur du 1er cycle (jusqu'au BAC+3)................................☐5
- Diplôme de l'enseignement supérieur du 2ème ou du 3ème cycle, diplômés des grandes écoles….......☐6

9. Quelle est votre **situation professionnelle actuelle** ?
(une seule réponse possible)

- Agriculteur ....☐01
- Artisan, commerçant, chef d'entreprise ....☐02
- Cadre, profession intellectuelle supérieure....☐03
- Profession intermédiaire.............................☐04
- Employé ....☐05
- Ouvrier..☐06

Passez à **10.**

- Retraité..☐07
- À la recherche d'un emploi.........................☐08
- Élève ou étudiant ....☐09
- Autre sans activité (personne au foyer…) ....☐10

Passez à **11.**

(Si vous exercez actuellement une profession)

10. Dans quel **secteur d'activité** travaillez-vous ?
(une seule réponse possible)

- Agriculture, sylviculture, pêche...................☐01
- Industrie agricole et alimentaire ....☐02
- Autre industrie..☐03
- Énergie ....☐04
- Construction ....☐05
- Commerce..☐06
- Activités financières et immobilières ....☐07
- Services aux entreprises ....☐08
- Services aux personnes.............................☐09
- Éducation, santé, action social...................☐10
- Administration ....☐11

Quelques informations sur les activités de l'enfant qui participe à l'enquête

11. L'enfant est-t-il scolarisé ?

- Oui .... ☐1
- Non .... ☐2

*Si votre enfant n'est **pas scolarisé**, répondez aux **questions 12 à 15***
*Si votre enfant est **scolarisé**, passez **directement à la question 16**.*

➛ Votre enfant n'est pas encore scolarisé :

12. Votre enfant est-il habituellement gardé **à la maison ou dans la famille** ?

- Oui.......................... ☐1
- Non......................... ☐2

13. Votre enfant est-il habituellement gardé **au domicile d'une assistante maternelle** ?

- Oui.......................... ☐1 ➛ *Passez à* **13a** et **13b.**
- Non......................... ☐2 ➛ *Passez à* **14.**

13a. En moyenne et sans compter votre enfant, combien d'enfants y a-t-il chez l'assistante maternelle :

☐ enfant(s)

13b. L'assistante maternelle accueille-t-elle l'après midi ou le soir des enfants scolarisés ?

- Oui.......................... ☐1
- Non......................... ☐2

14. Votre enfant est-il habituellement gardé **en crèche** ?

- Oui.......................... ☐1 ➛ *Passez à* **14a.**
- Non......................... ☐2 ➛ *Passez à* **15.**

14a. Dans la crèche :
il y a en moyenne combien d'enfants ?

- Moins de 20 enfants .... ☐1
- Entre 20 et 50 enfants.................................... ☐2
- Plus de 50 enfants .... ☐3

15. Si votre enfant n'est pas gardé en crèche :
à quelle fréquence votre enfant va-t-il en halte-garderie ?

- Plus d'une fois par semaine........................... ☐1
- Une fois par semaine .... ☐2
- Très rarement .... ☐3
- Jamais... ☐4

Allez directement en question 18.

➛ Votre enfant est scolarisé :

16. Combien y-a-t'il d'enfants dans sa classe ?

- il y a moins de 20 enfants dans la classe ..... ☐1
- entre 20 et 30 enfants dans la classe............. ☐2
- plus de 30 enfants dans la classe .... ☐3

17. Lorsqu'il va à l'école, votre enfant …?
(une seule réponse possible)

- ne mange jamais à la cantine........................ ☐1
- mange occasionnellement à la cantine .... ☐2
- est demi-pensionnaire .... ☐3
- est interne .... ☐4

➛ Les loisirs de votre enfant (qu'il soit ou non scolarisé) :

18. Votre enfant a -t-il des activités dans un centre aéré ou un centre de loisirs ?

- Oui .... ☐1 ➛ *Passez à* **18a** et **18b.**
- Non .... ☐2 ➛ *Passez à la page suivante*

18a. Le mercredi ou la samedi, durant les périodes scolaires, votre enfant a -t-il des activités dans un centre aéré ou un centre de loisirs… ?

- Toutes les semaines .... ☐1
- Occasionnellement... ☐2
- Jamais... ☐3

18b. Durant les vacances, votre enfant a -t-il des activités dans un centre aéré ou un centre de loisirs … ?

- Plus de 5 semaines par an .... ☐1
- Entre 5 et 2 semaines par an.......................... ☐2
- Moins de 2 semaines par an .... ☐3
- Jamais... ☐4

Guide de remplissage du carnet

Nous vous prions d'indiquer dans ce journal toutes les personnes avec qui l'enfant a un **contact direct** et qu'il a rencontrées durant **les deux journées retenues**.

① **L'enquête concerne les contacts directs**

Qu'est ce qu'un contact direct ?

- Un contact veut dire que l'enfant a **parlé avec quelqu'un en sa présence physique et à une distance inférieure de 2 mètres**.
  - Les contacts par téléphone ou internet sont exclus,
  - les contacts ayant donné à une discussion non rapprochée (plus de 2 mètres) ne doivent pas être pris en compte.
- Un contact peut aussi être physique: **toucher la peau** de l'autre personne (se donner ou se serrer la main, s'embrasser, se donner l'accolade…).

On ne retient pas les contacts avec des animaux.

② **Une ligne par personne contactée**

Il faut utiliser une seule ligne par personne contactée.

Si l'enfant a rencontré la même personne plusieurs fois dans la même journée, ne remplissez qu'une seule ligne en estimant au total combien de temps il a passé avec cette personne dans la journée.

Exemple 1 : *Ce matin, vous avez accompagné votre fille à l'école en prenant le bus. Sa maîtresse était dans le bus et votre fille lui a dit bonjour en s'approchant d'elle. En fin de journée, en partant de l'école, votre fille a un peu discuté avec sa maîtresse. Sa maîtresse vous a expliqué qu'à la récréation elle a mis un pansement au genou de votre fille. Vous connaissez l'âge exact de la maîtresse qui a 26 ans*

***Ce qu'il faut noter dans le carnet :** il faut indiquer sur une même ligne l'ensemble des contacts que votre enfant a eu avec sa maîtresse. Ne pas oublier de consigner **tous les lieux** où ont eu lieu les contacts : transport en commun (bus) et école. Additionner la durée totale des contacts : 20 minutes.*

③ **Conseils généraux sur le remplissage du questionnaire**

La réponse au questionnaire sera plus facile si vous (ou votre enfant / ou la personne qui sera avec lui) prenez des notes au fur et à mesure au cours de la journée (toutes les 2 heures ou après les repas par exemple). Vous pouvez aussi vous appuyer sur son rythme habituel.

Vous pouvez décrire ses contacts directs **par ordre chronologique**, en commençant par la personne qu'il a rencontrée en premier lors de la journée et en continuant avec toutes les autres personnes dont vous pouvez ou dont il peut se souvenir en fonction des activités de la journée.

④ **Les difficultés possibles**

Je ne connais pas l'âge de la personne avec qui l'enfant a eu un contact direct ?

Donnez **une estimation de l'âge de la personne** avec qui il a eu un contact.

Exemple 2 : *Avec votre fils, vous êtes allés faire des courses. La vendeuse qui avait une trentaine d'années lui a touché la main en lui tendant un produit. Vous allez rarement dans ce magasin.*

***Ce qu'il faut noter dans le carnet :** une fourchette pour l'âge de la vendeuse (30-35) et son sexe, le lieu (autre lieu clos), la fréquence des rencontres (la première fois), la vendeuse a touché la peau de votre fils, le contact a duré moins de 5 minutes.*

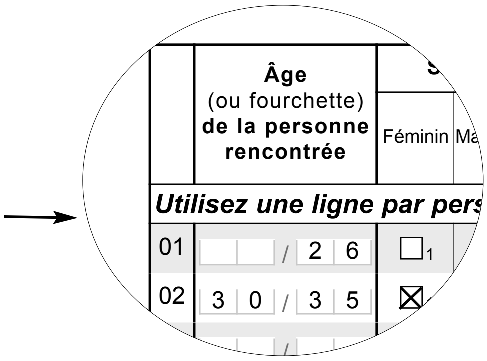

Que dois-je faire si l'enfant à eu plusieurs contacts directs avec la même personne pendant la journée ?

Utilisez une seule ligne et estimez le temps total qu'il a passé avec cette personne lors de la journée attribuée.

Dans l'exemple 1 : Tous les contacts avec la maîtresse (dans la même journée) sont notés sur une seule ligne (bonjour du matin, pansement, et discussion du soir soit 20 minutes de contact au total)

A noter que la durée totale des contacts est inférieure au temps que votre enfant a passé avec sa maîtresse : environ 6h mais au fond de la classe donc pas en situation de contact.

⑤ **Une fois le questionnaire rempli**

Quand vous avez décrit le dernier contact direct de l'enfant, nous vous conseillons de réfléchir encore une fois afin de vérifier avec lui que vous n'avez pas oublié une activité ou un contact.
Son agenda pourra être utile.

N'oubliez pas de décrire les contacts que vous avez avec l'enfant.

Au moment de remplir les grilles, n'hésitez pas à vous référez au document 'Aide au remplissage' - tous les exemples sont illustrés et les consignes rappelées - nous espérons que cela vous aidera.

Jour 1 : |__|__| |__|__|

Carnet des contacts de l'enfant désigné pour participer à l'enquête

	**Âge** (ou fourchette) **de la personne rencontrée par l'enfant**	**Sexe**		**Lieu(x) de contacts** noter <u>tous les lieux</u> où la personne a été en contact avec votre enfant							**À quelle fréquence votre enfant rencontre-il cette personne ?**					**A-t-elle touché sa peau ?**		**Durée totale passée avec la personne**				
		Féminin	Masculin	Domicile, véhicule ou autres lieux privatifs	Crèche, école, collège	Centre scolaire ou de loisirs avec hébergement (Colonie, internat, …)	Chez des proches en lieux clos	Autre lieux clos (restaurant, commerce, …)	Transport collectif	Lieux ouverts (parc, rue…)	(presque) chaque jour	Quelques fois par semaine	Quelques fois par mois	Quelques fois par an ou moins souvent	1ère fois	Oui	Non	Moins de 5 min	5 -15 min	15 min - 1 h	1-4 h	4H ou plus
Utilisez une ligne par personne rencontrée et avec laquelle votre enfant a eu au moins un 'contact'																						
01	/	☐1	☐2	☐1	☐2	☐3	☐4	☐5	☐6	☐7	☐1	☐2	☐3	☐4	☐5	☐1	☐2	☐1	☐2	☐3	☐4	☐5
02	/	☐1	☐2	☐1	☐2	☐3	☐4	☐5	☐6	☐7	☐1	☐2	☐3	☐4	☐5	☐1	☐2	☐1	☐2	☐3	☐4	☐5
03	/	☐1	☐2	☐1	☐2	☐3	☐4	☐5	☐6	☐7	☐1	☐2	☐3	☐4	☐5	☐1	☐2	☐1	☐2	☐3	☐4	☐5
04	/	☐1	☐2	☐1	☐2	☐3	☐4	☐5	☐6	☐7	☐1	☐2	☐3	☐4	☐5	☐1	☐2	☐1	☐2	☐3	☐4	☐5
05	/	☐1	☐2	☐1	☐2	☐3	☐4	☐5	☐6	☐7	☐1	☐2	☐3	☐4	☐5	☐1	☐2	☐1	☐2	☐3	☐4	☐5
06	/	☐1	☐2	☐1	☐2	☐3	☐4	☐5	☐6	☐7	☐1	☐2	☐3	☐4	☐5	☐1	☐2	☐1	☐2	☐3	☐4	☐5
07	/	☐1	☐2	☐1	☐2	☐3	☐4	☐5	☐6	☐7	☐1	☐2	☐3	☐4	☐5	☐1	☐2	☐1	☐2	☐3	☐4	☐5
08	/	☐1	☐2	☐1	☐2	☐3	☐4	☐5	☐6	☐7	☐1	☐2	☐3	☐4	☐5	☐1	☐2	☐1	☐2	☐3	☐4	☐5
09	/	☐1	☐2	☐1	☐2	☐3	☐4	☐5	☐6	☐7	☐1	☐2	☐3	☐4	☐5	☐1	☐2	☐1	☐2	☐3	☐4	☐5
10	/	☐1	☐2	☐1	☐2	☐3	☐4	☐5	☐6	☐7	☐1	☐2	☐3	☐4	☐5	☐1	☐2	☐1	☐2	☐3	☐4	☐5
11	/	☐1	☐2	☐1	☐2	☐3	☐4	☐5	☐6	☐7	☐1	☐2	☐3	☐4	☐5	☐1	☐2	☐1	☐2	☐3	☐4	☐5
12	/	☐1	☐2	☐1	☐2	☐3	☐4	☐5	☐6	☐7	☐1	☐2	☐3	☐4	☐5	☐1	☐2	☐1	☐2	☐3	☐4	☐5
13	/	☐1	☐2	☐1	☐2	☐3	☐4	☐5	☐6	☐7	☐1	☐2	☐3	☐4	☐5	☐1	☐2	☐1	☐2	☐3	☐4	☐5
14	/	☐1	☐2	☐1	☐2	☐3	☐4	☐5	☐6	☐7	☐1	☐2	☐3	☐4	☐5	☐1	☐2	☐1	☐2	☐3	☐4	☐5
15	/	☐1	☐2	☐1	☐2	☐3	☐4	☐5	☐6	☐7	☐1	☐2	☐3	☐4	☐5	☐1	☐2	☐1	☐2	☐3	☐4	☐5
16	/	☐1	☐2	☐1	☐2	☐3	☐4	☐5	☐6	☐7	☐1	☐2	☐3	☐4	☐5	☐1	☐2	☐1	☐2	☐3	☐4	☐5
17	/	☐1	☐2	☐1	☐2	☐3	☐4	☐5	☐6	☐7	☐1	☐2	☐3	☐4	☐5	☐1	☐2	☐1	☐2	☐3	☐4	☐5
18	/	☐1	☐2	☐1	☐2	☐3	☐4	☐5	☐6	☐7	☐1	☐2	☐3	☐4	☐5	☐1	☐2	☐1	☐2	☐3	☐4	☐5
19	/	☐1	☐2	☐1	☐2	☐3	☐4	☐5	☐6	☐7	☐1	☐2	☐3	☐4	☐5	☐1	☐2	☐1	☐2	☐3	☐4	☐5
20	/	☐1	☐2	☐1	☐2	☐3	☐4	☐5	☐6	☐7	☐1	☐2	☐3	☐4	☐5	☐1	☐2	☐1	☐2	☐3	☐4	☐5

Jour 1 *(suite…)*

	Âge (ou fourchette) **de la personne rencontrée par l'enfant**	**Sexe**		**Lieu(x) de contacts** noter <u>tous les lieux</u> où la personne a été en contact avec votre enfant							**À quelle fréquence votre enfant rencontre-il cette personne ?**					**A-t-elle touché sa peau ?**		**Durée totale passée avec la personne**				
		Féminin	Masculin	Domicile, véhicule ou autres lieux privatifs	Crèche, école, collège	Centre scolaire ou de loisirs avec hébergement (Colonie, internat, …)	Chez des proches en lieux clos	Autre lieux clos (restaurant, commerce, …)	Transport collectif	Lieux ouverts (parc, rue…)	(presque) chaque jour	Quelques fois par semaine	Quelques fois par mois	Quelques fois par an ou moins souvent	1ère fois	Oui	Non	Moins de 5 min	5 -15 min	15 min - 1 h	1-4 h	4H ou plus
21	__ __ / __ __	☐1	☐2	☐1	☐2	☐3	☐4	☐5	☐6	☐7	☐1	☐2	☐3	☐4	☐5	☐1	☐2	☐1	☐2	☐3	☐4	☐5
22	__ __ / __ __	☐1	☐2	☐1	☐2	☐3	☐4	☐5	☐6	☐7	☐1	☐2	☐3	☐4	☐5	☐1	☐2	☐1	☐2	☐3	☐4	☐5
23	__ __ / __ __	☐1	☐2	☐1	☐2	☐3	☐4	☐5	☐6	☐7	☐1	☐2	☐3	☐4	☐5	☐1	☐2	☐1	☐2	☐3	☐4	☐5
24	__ __ / __ __	☐1	☐2	☐1	☐2	☐3	☐4	☐5	☐6	☐7	☐1	☐2	☐3	☐4	☐5	☐1	☐2	☐1	☐2	☐3	☐4	☐5
25	__ __ / __ __	☐1	☐2	☐1	☐2	☐3	☐4	☐5	☐6	☐7	☐1	☐2	☐3	☐4	☐5	☐1	☐2	☐1	☐2	☐3	☐4	☐5
26	__ __ / __ __	☐1	☐2	☐1	☐2	☐3	☐4	☐5	☐6	☐7	☐1	☐2	☐3	☐4	☐5	☐1	☐2	☐1	☐2	☐3	☐4	☐5
27	__ __ / __ __	☐1	☐2	☐1	☐2	☐3	☐4	☐5	☐6	☐7	☐1	☐2	☐3	☐4	☐5	☐1	☐2	☐1	☐2	☐3	☐4	☐5
28	__ __ / __ __	☐1	☐2	☐1	☐2	☐3	☐4	☐5	☐6	☐7	☐1	☐2	☐3	☐4	☐5	☐1	☐2	☐1	☐2	☐3	☐4	☐5
29	__ __ / __ __	☐1	☐2	☐1	☐2	☐3	☐4	☐5	☐6	☐7	☐1	☐2	☐3	☐4	☐5	☐1	☐2	☐1	☐2	☐3	☐4	☐5
30	__ __ / __ __	☐1	☐2	☐1	☐2	☐3	☐4	☐5	☐6	☐7	☐1	☐2	☐3	☐4	☐5	☐1	☐2	☐1	☐2	☐3	☐4	☐5
31	__ __ / __ __	☐1	☐2	☐1	☐2	☐3	☐4	☐5	☐6	☐7	☐1	☐2	☐3	☐4	☐5	☐1	☐2	☐1	☐2	☐3	☐4	☐5
32	__ __ / __ __	☐1	☐2	☐1	☐2	☐3	☐4	☐5	☐6	☐7	☐1	☐2	☐3	☐4	☐5	☐1	☐2	☐1	☐2	☐3	☐4	☐5
33	__ __ / __ __	☐1	☐2	☐1	☐2	☐3	☐4	☐5	☐6	☐7	☐1	☐2	☐3	☐4	☐5	☐1	☐2	☐1	☐2	☐3	☐4	☐5
34	__ __ / __ __	☐1	☐2	☐1	☐2	☐3	☐4	☐5	☐6	☐7	☐1	☐2	☐3	☐4	☐5	☐1	☐2	☐1	☐2	☐3	☐4	☐5
35	__ __ / __ __	☐1	☐2	☐1	☐2	☐3	☐4	☐5	☐6	☐7	☐1	☐2	☐3	☐4	☐5	☐1	☐2	☐1	☐2	☐3	☐4	☐5
36	__ __ / __ __	☐1	☐2	☐1	☐2	☐3	☐4	☐5	☐6	☐7	☐1	☐2	☐3	☐4	☐5	☐1	☐2	☐1	☐2	☐3	☐4	☐5
37	__ __ / __ __	☐1	☐2	☐1	☐2	☐3	☐4	☐5	☐6	☐7	☐1	☐2	☐3	☐4	☐5	☐1	☐2	☐1	☐2	☐3	☐4	☐5
38	__ __ / __ __	☐1	☐2	☐1	☐2	☐3	☐4	☐5	☐6	☐7	☐1	☐2	☐3	☐4	☐5	☐1	☐2	☐1	☐2	☐3	☐4	☐5
39	__ __ / __ __	☐1	☐2	☐1	☐2	☐3	☐4	☐5	☐6	☐7	☐1	☐2	☐3	☐4	☐5	☐1	☐2	☐1	☐2	☐3	☐4	☐5
40	__ __ / __ __	☐1	☐2	☐1	☐2	☐3	☐4	☐5	☐6	☐7	☐1	☐2	☐3	☐4	☐5	☐1	☐2	☐1	☐2	☐3	☐4	☐5

Jour 2 : ☐☐ ☐☐

Carnet des contacts de l'enfant désigné pour participer à l'enquête

	**Âge** (ou fourchette) **de la personne rencontrée par l'enfant**	**Sexe**		**Lieu(x) de contacts** noter tous les lieux où la personne a été en contact avec votre enfant							**À quelle fréquence votre enfant rencontre-il cette personne ?**					**A-t-elle touché sa peau ?**		**Durée totale passée avec la personne**				
		Féminin	Masculin	Domicile, véhicule ou autres lieux privatifs	Crèche, école, collège	Centre scolaire ou de loisirs avec hébergement (Colonie, internat, …)	Chez des proches en lieux clos	Autre lieux clos (restaurant, commerce, …)	Transport collectif	Lieux ouverts (parc, rue…)	(presque) chaque jour	Quelques fois par semaine	Quelques fois par mois	Quelques fois par an ou moins souvent	1ère fois	Oui	Non	Moins de 5 min	5 -15 min	15 min - 1 h	1-4 h	4H ou plus
Utilisez une ligne par personne rencontrée et avec laquelle votre enfant a eu au moins un 'contact'																						
**01**	☐☐ / ☐☐	☐1	☐2	☐1	☐2	☐3	☐4	☐5	☐6	☐7	☐1	☐2	☐3	☐4	☐5	☐1	☐2	☐1	☐2	☐3	☐4	☐5
**02**	☐☐ / ☐☐	☐1	☐2	☐1	☐2	☐3	☐4	☐5	☐6	☐7	☐1	☐2	☐3	☐4	☐5	☐1	☐2	☐1	☐2	☐3	☐4	☐5
**03**	☐☐ / ☐☐	☐1	☐2	☐1	☐2	☐3	☐4	☐5	☐6	☐7	☐1	☐2	☐3	☐4	☐5	☐1	☐2	☐1	☐2	☐3	☐4	☐5
**04**	☐☐ / ☐☐	☐1	☐2	☐1	☐2	☐3	☐4	☐5	☐6	☐7	☐1	☐2	☐3	☐4	☐5	☐1	☐2	☐1	☐2	☐3	☐4	☐5
**05**	☐☐ / ☐☐	☐1	☐2	☐1	☐2	☐3	☐4	☐5	☐6	☐7	☐1	☐2	☐3	☐4	☐5	☐1	☐2	☐1	☐2	☐3	☐4	☐5
**06**	☐☐ / ☐☐	☐1	☐2	☐1	☐2	☐3	☐4	☐5	☐6	☐7	☐1	☐2	☐3	☐4	☐5	☐1	☐2	☐1	☐2	☐3	☐4	☐5
**07**	☐☐ / ☐☐	☐1	☐2	☐1	☐2	☐3	☐4	☐5	☐6	☐7	☐1	☐2	☐3	☐4	☐5	☐1	☐2	☐1	☐2	☐3	☐4	☐5
**08**	☐☐ / ☐☐	☐1	☐2	☐1	☐2	☐3	☐4	☐5	☐6	☐7	☐1	☐2	☐3	☐4	☐5	☐1	☐2	☐1	☐2	☐3	☐4	☐5
**09**	☐☐ / ☐☐	☐1	☐2	☐1	☐2	☐3	☐4	☐5	☐6	☐7	☐1	☐2	☐3	☐4	☐5	☐1	☐2	☐1	☐2	☐3	☐4	☐5
**10**	☐☐ / ☐☐	☐1	☐2	☐1	☐2	☐3	☐4	☐5	☐6	☐7	☐1	☐2	☐3	☐4	☐5	☐1	☐2	☐1	☐2	☐3	☐4	☐5
**11**	☐☐ / ☐☐	☐1	☐2	☐1	☐2	☐3	☐4	☐5	☐6	☐7	☐1	☐2	☐3	☐4	☐5	☐1	☐2	☐1	☐2	☐3	☐4	☐5
**12**	☐☐ / ☐☐	☐1	☐2	☐1	☐2	☐3	☐4	☐5	☐6	☐7	☐1	☐2	☐3	☐4	☐5	☐1	☐2	☐1	☐2	☐3	☐4	☐5
**13**	☐☐ / ☐☐	☐1	☐2	☐1	☐2	☐3	☐4	☐5	☐6	☐7	☐1	☐2	☐3	☐4	☐5	☐1	☐2	☐1	☐2	☐3	☐4	☐5
**14**	☐☐ / ☐☐	☐1	☐2	☐1	☐2	☐3	☐4	☐5	☐6	☐7	☐1	☐2	☐3	☐4	☐5	☐1	☐2	☐1	☐2	☐3	☐4	☐5
**15**	☐☐ / ☐☐	☐1	☐2	☐1	☐2	☐3	☐4	☐5	☐6	☐7	☐1	☐2	☐3	☐4	☐5	☐1	☐2	☐1	☐2	☐3	☐4	☐5
**16**	☐☐ / ☐☐	☐1	☐2	☐1	☐2	☐3	☐4	☐5	☐6	☐7	☐1	☐2	☐3	☐4	☐5	☐1	☐2	☐1	☐2	☐3	☐4	☐5
**17**	☐☐ / ☐☐	☐1	☐2	☐1	☐2	☐3	☐4	☐5	☐6	☐7	☐1	☐2	☐3	☐4	☐5	☐1	☐2	☐1	☐2	☐3	☐4	☐5
**18**	☐☐ / ☐☐	☐1	☐2	☐1	☐2	☐3	☐4	☐5	☐6	☐7	☐1	☐2	☐3	☐4	☐5	☐1	☐2	☐1	☐2	☐3	☐4	☐5
**19**	☐☐ / ☐☐	☐1	☐2	☐1	☐2	☐3	☐4	☐5	☐6	☐7	☐1	☐2	☐3	☐4	☐5	☐1	☐2	☐1	☐2	☐3	☐4	☐5
**20**	☐☐ / ☐☐	☐1	☐2	☐1	☐2	☐3	☐4	☐5	☐6	☐7	☐1	☐2	☐3	☐4	☐5	☐1	☐2	☐1	☐2	☐3	☐4	☐5

Jour 2 *(suite…)*

	Âge (ou fourchette) **de la personne rencontrée par l'enfant**	**Sexe**		**Lieu(x) de contacts** noter <u>tous les lieux</u> où la personne a été en contact avec votre enfant							**À quelle fréquence votre enfant rencontre-il cette personne ?**					**A-t-elle touché sa peau ?**		**Durée totale passée avec la personne**				
		Féminin	Masculin	Domicile, véhicule ou autres lieux privatifs	Crèche, école, collège	Centre scolaire ou de loisirs avec hébergement (Colonie, internat, …)	Chez des proches en lieux clos	Autre lieux clos (restaurant, commerce, …)	Transport collectif	Lieux ouverts (parc, rue…)	(presque) chaque jour	Quelques fois par semaine	Quelques fois par mois	Quelques fois par an ou moins souvent	1ère fois	Oui	Non	Moins de 5 min	5 -15 min	15 min - 1 h	1-4 h	4H ou plus
**21**	/	☐1	☐2	☐1	☐2	☐3	☐4	☐5	☐6	☐7	☐1	☐2	☐3	☐4	☐5	☐1	☐2	☐1	☐2	☐3	☐4	☐5
**22**	/	☐1	☐2	☐1	☐2	☐3	☐4	☐5	☐6	☐7	☐1	☐2	☐3	☐4	☐5	☐1	☐2	☐1	☐2	☐3	☐4	☐5
**23**	/	☐1	☐2	☐1	☐2	☐3	☐4	☐5	☐6	☐7	☐1	☐2	☐3	☐4	☐5	☐1	☐2	☐1	☐2	☐3	☐4	☐5
**24**	/	☐1	☐2	☐1	☐2	☐3	☐4	☐5	☐6	☐7	☐1	☐2	☐3	☐4	☐5	☐1	☐2	☐1	☐2	☐3	☐4	☐5
**25**	/	☐1	☐2	☐1	☐2	☐3	☐4	☐5	☐6	☐7	☐1	☐2	☐3	☐4	☐5	☐1	☐2	☐1	☐2	☐3	☐4	☐5
**26**	/	☐1	☐2	☐1	☐2	☐3	☐4	☐5	☐6	☐7	☐1	☐2	☐3	☐4	☐5	☐1	☐2	☐1	☐2	☐3	☐4	☐5
**27**	/	☐1	☐2	☐1	☐2	☐3	☐4	☐5	☐6	☐7	☐1	☐2	☐3	☐4	☐5	☐1	☐2	☐1	☐2	☐3	☐4	☐5
**28**	/	☐1	☐2	☐1	☐2	☐3	☐4	☐5	☐6	☐7	☐1	☐2	☐3	☐4	☐5	☐1	☐2	☐1	☐2	☐3	☐4	☐5
**29**	/	☐1	☐2	☐1	☐2	☐3	☐4	☐5	☐6	☐7	☐1	☐2	☐3	☐4	☐5	☐1	☐2	☐1	☐2	☐3	☐4	☐5
**30**	/	☐1	☐2	☐1	☐2	☐3	☐4	☐5	☐6	☐7	☐1	☐2	☐3	☐4	☐5	☐1	☐2	☐1	☐2	☐3	☐4	☐5
**31**	/	☐1	☐2	☐1	☐2	☐3	☐4	☐5	☐6	☐7	☐1	☐2	☐3	☐4	☐5	☐1	☐2	☐1	☐2	☐3	☐4	☐5
**32**	/	☐1	☐2	☐1	☐2	☐3	☐4	☐5	☐6	☐7	☐1	☐2	☐3	☐4	☐5	☐1	☐2	☐1	☐2	☐3	☐4	☐5
**33**	/	☐1	☐2	☐1	☐2	☐3	☐4	☐5	☐6	☐7	☐1	☐2	☐3	☐4	☐5	☐1	☐2	☐1	☐2	☐3	☐4	☐5
**34**	/	☐1	☐2	☐1	☐2	☐3	☐4	☐5	☐6	☐7	☐1	☐2	☐3	☐4	☐5	☐1	☐2	☐1	☐2	☐3	☐4	☐5
**35**	/	☐1	☐2	☐1	☐2	☐3	☐4	☐5	☐6	☐7	☐1	☐2	☐3	☐4	☐5	☐1	☐2	☐1	☐2	☐3	☐4	☐5
**36**	/	☐1	☐2	☐1	☐2	☐3	☐4	☐5	☐6	☐7	☐1	☐2	☐3	☐4	☐5	☐1	☐2	☐1	☐2	☐3	☐4	☐5
**37**	/	☐1	☐2	☐1	☐2	☐3	☐4	☐5	☐6	☐7	☐1	☐2	☐3	☐4	☐5	☐1	☐2	☐1	☐2	☐3	☐4	☐5
**38**	/	☐1	☐2	☐1	☐2	☐3	☐4	☐5	☐6	☐7	☐1	☐2	☐3	☐4	☐5	☐1	☐2	☐1	☐2	☐3	☐4	☐5
**39**	/	☐1	☐2	☐1	☐2	☐3	☐4	☐5	☐6	☐7	☐1	☐2	☐3	☐4	☐5	☐1	☐2	☐1	☐2	☐3	☐4	☐5
**40**	/	☐1	☐2	☐1	☐2	☐3	☐4	☐5	☐6	☐7	☐1	☐2	☐3	☐4	☐5	☐1	☐2	☐1	☐2	☐3	☐4	☐5

Notes

Un événement particulier s'est déroulé pendant les jours d'enquête, vous vous êtes posé des questions lors du remplissage, vous souhaitez nous apporter des précisions… merci d'écrire vos commentaires ci-dessous…

Pour vous aider

N'hésitez pas à relire **les explications qui se trouvent en page 3 de votre carnet**, ou à vous reporter **aux exemples qui figurent sur la feuille 'Aide au remplissage'**.

Un N° de téléphone est à votre disposition : vous pouvez nous contacter aux heures de bureau si vous avez des questions ou des informations à nous communiquer.

Pour de plus amples informations, vous pouvez aussi nous téléphoner ou nous écrire.

Email : **enquete.contacts@ipsos.com**

Tél : **08 00 97 07 32**

MERCI

d'avoir participé à cette enquête, aux noms d'Ipsos et
de l'équipe de recherche en charge de ce projet.
