# Supplementary figures and images for: The French Connection: The First Large Population-Based Contact Survey in France Relevant for the Spread of Infectious Diseases
Source: PLoS One. 2015 Jul 15;10(7):e0133203. doi: 10.1371/journal.pone.0133203 (PMC4503306; doi:10.1371/journal.pone.0133203)

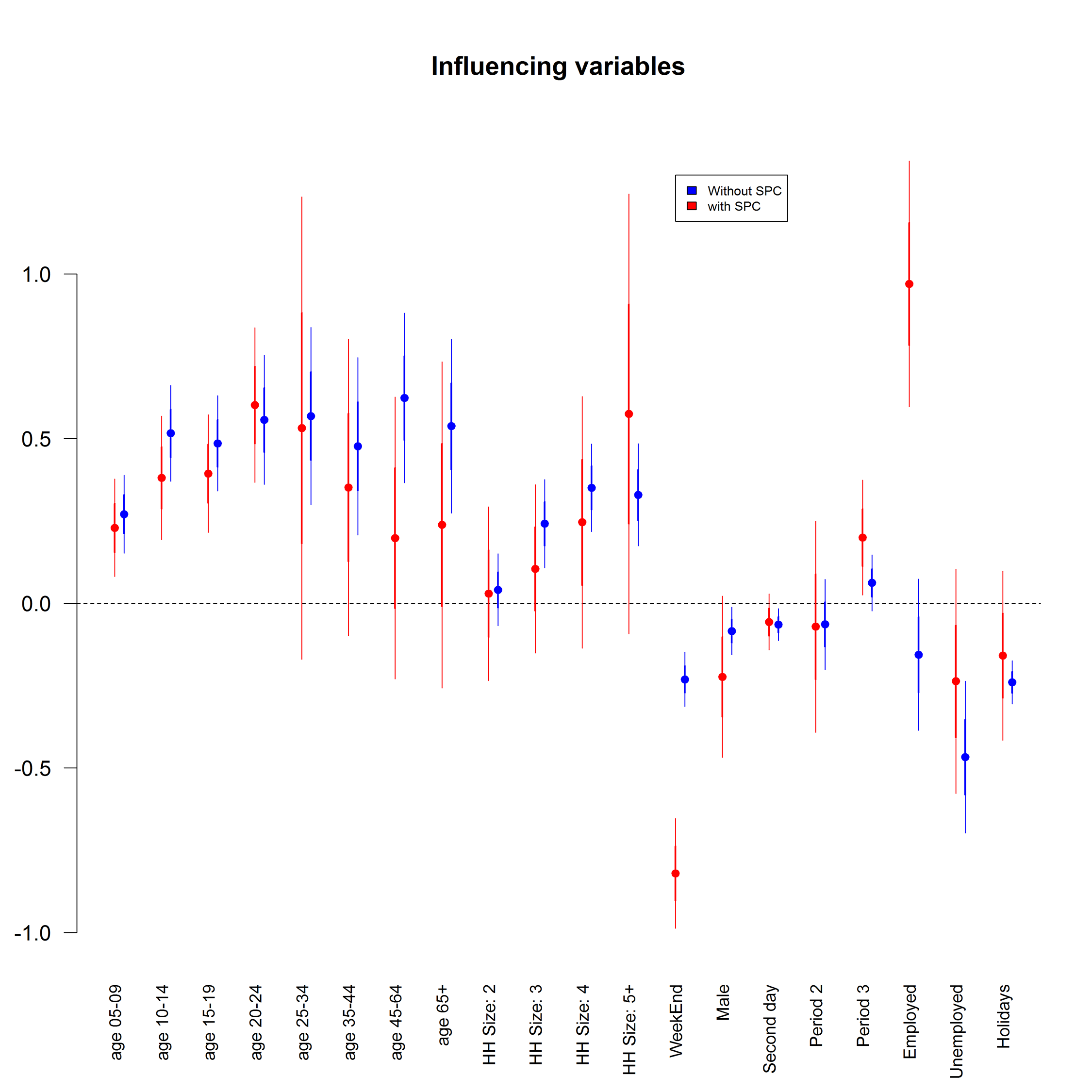

Supplement: S2 Fig — (TIF) [file pone.0133203.s002.tif]

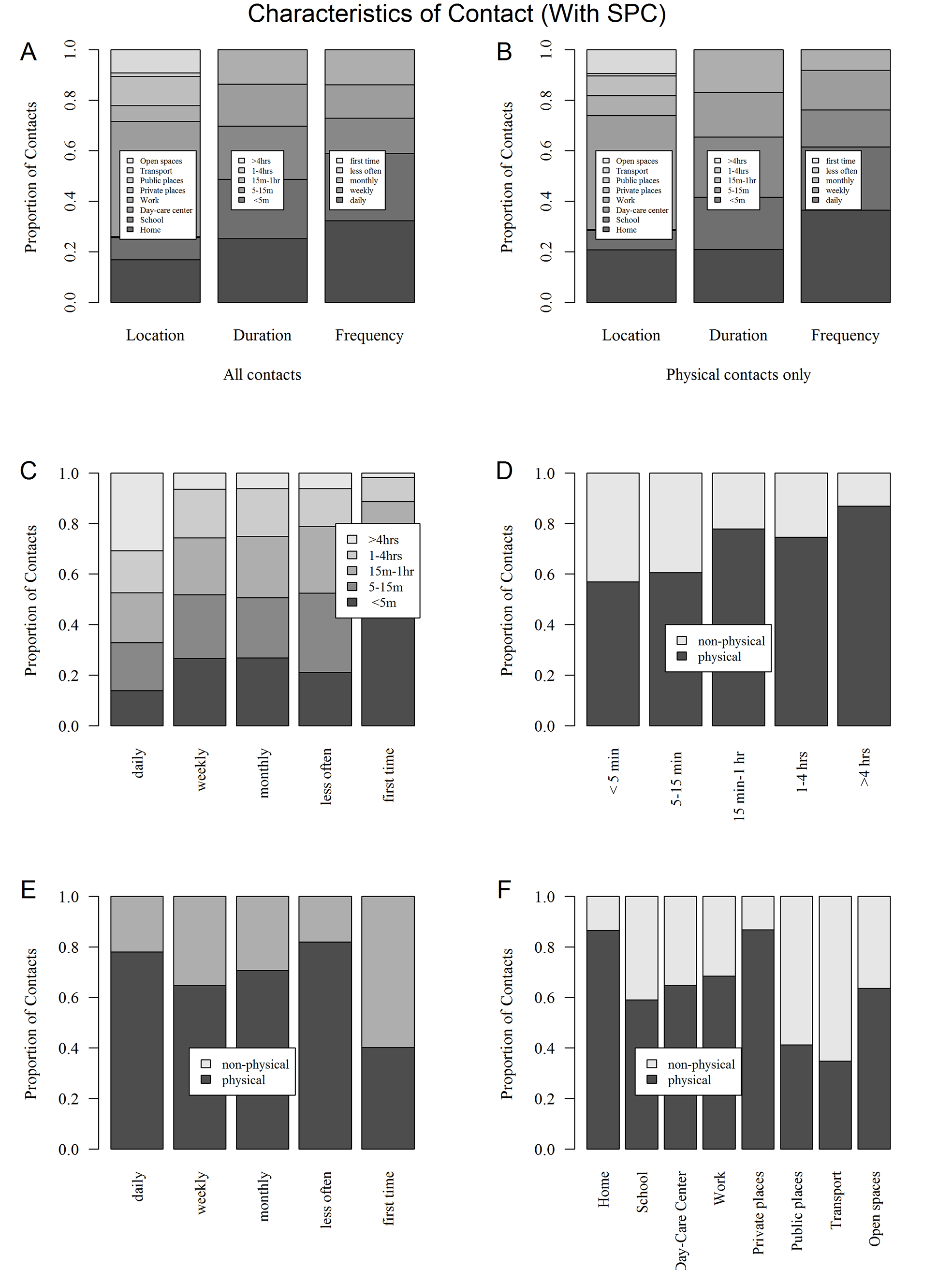

Supplement: S4 Fig — (TIF) [file pone.0133203.s004.tif]
