# Supplementary material for: The French Connection: The First Large Population-Based Contact Survey in France Relevant for the Spread of Infectious Diseases
Source: PLoS One. 2015 Jul 15;10(7):e0133203. doi: 10.1371/journal.pone.0133203 (PMC4503306; doi:10.1371/journal.pone.0133203)
Supplement: S3 Fig — (PDF) [file pone.0133203.s003.pdf]

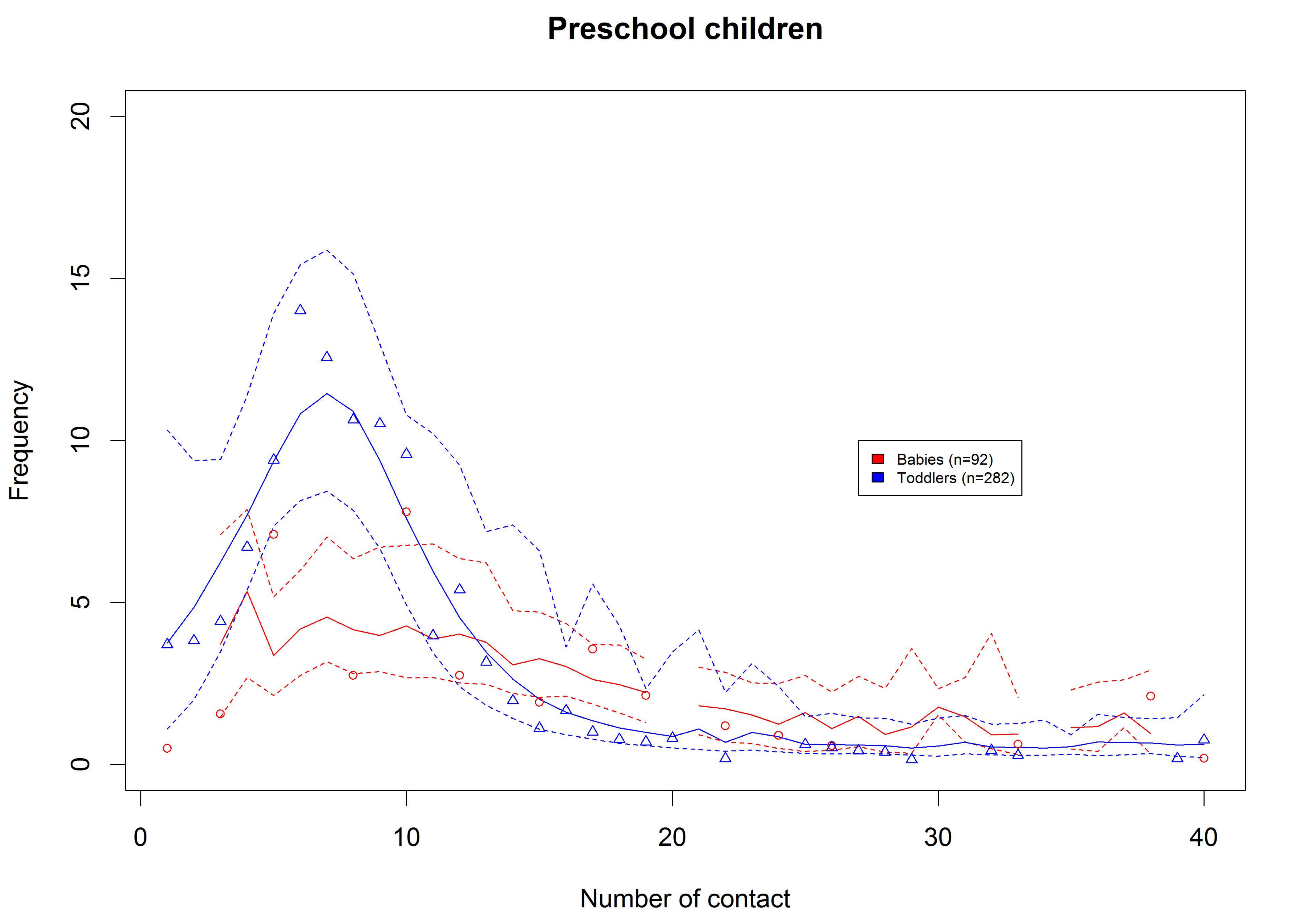

Preschool children
Frequency
Number of contact
Babies (n=92)
Toddlers (n=282)
0
5
10
15
20
0
10
20
30
40

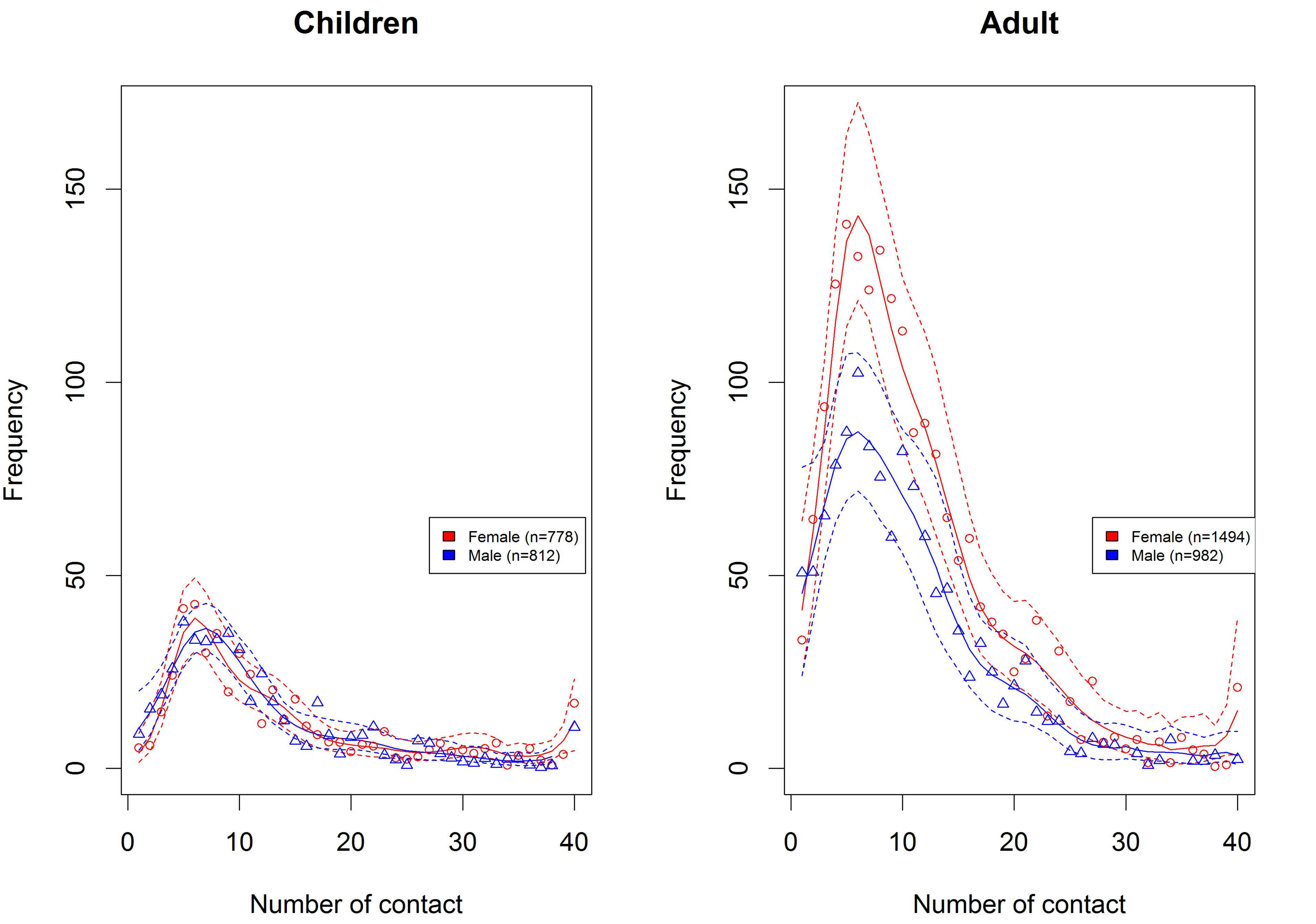
Children
Adult
Frequency
Number of contact
Female (n=778)
Male (n=812)
Female (n=1494)
Male (n=982)
0
50
100
150
0
10
20
30
40

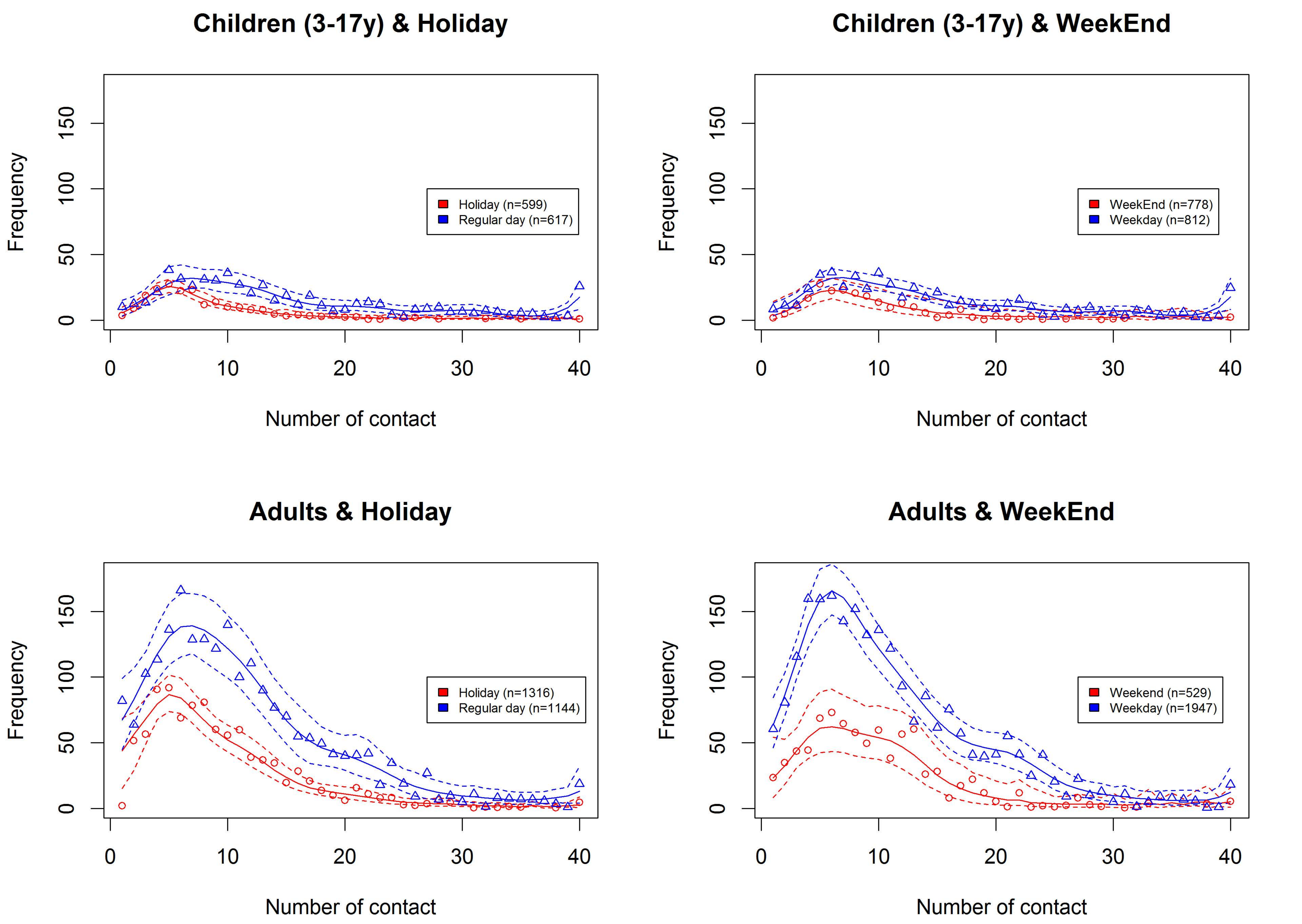
Children (3-17y) & Holiday
Holiday (n=599)
Regular day (n=617)
Children (3-17y) & WeekEnd
WeekEnd (n=778)
Weekday (n=812)
Adults & Holiday
Holiday (n=1316)
Regular day (n=1144)
Adults & WeekEnd
Weekend (n=529)
Weekday (n=1947)
Frequency
Number of contact
0
50
100
150
0
10
20
30
40
