# Supplementary material for: The French Connection: The First Large Population-Based Contact Survey in France Relevant for the Spread of Infectious Diseases
Source: PLoS One. 2015 Jul 15;10(7):e0133203. doi: 10.1371/journal.pone.0133203 (PMC4503306; doi:10.1371/journal.pone.0133203)
Supplement: S1 Text — (DOCX) [file pone.0133203.s005.docx]

**S1 Text. Next Generation Matrix and R0**

We compared contact patterns not only graphically but also using the basic reproduction number R_0_, i.e. the mean number of secondary cases a typical single infectious case will cause in a population with no immunity to the disease. Diekmann et al (J Math Biol. 1990) showed that R_0_ can be defined as the dominant eigenvalue of the next-generation matrix when the entire population is susceptible. R_0_ has threshold value 1, in the sense that an epidemic will result from introduction of the infective agent when R_0_ > 1, while the number of new infections per day declines right after the introduction when R_0_ ≤ 1. Using the methodology by Hens et al (BMC Infectious diseases 2009), we considered the ratio of R_0_ estimated from different contact matrices. For comparing 2 contact matrices $C_{a}$ and $C_{b}$, the ratio is calculated according to:

$$\frac{R_{0, 1}}{R_{0,2}}=\frac{Max Eigen Value\left( \frac{N_{\left( a \right)}D}{L}q\times C_{1}(a,a') \right)}{Max Eigen Value\left( \frac{N_{\left( a \right)}D}{L}q\times C_{2}(a,a^{'}) \right)}$$

With population size N stratified by age, mean duration of infectiousness D and life expectancy L, the proportionality factor q measuring among other things the disease-specific infectivity and susceptibility and C the contact matrix. After cancelling the normalizing constant, the ratio relates only to contact data. Under the null hypothesis of equal contact matrices and assuming *q* to be constant, this ratio is expected to equal 1. For each comparison, we assess the significance of any deviation from the null hypothesis by calculating 95% confidence intervals based on a paired nonparametric bootstrap. We also calculate the expected age-specific relative incidence in the population during the exponential phase, as given by the leading eigenvector of the next-generation matrix.
