# Supplementary material for: The French Connection: The First Large Population-Based Contact Survey in France Relevant for the Spread of Infectious Diseases
Source: PLoS One. 2015 Jul 15;10(7):e0133203. doi: 10.1371/journal.pone.0133203 (PMC4503306; doi:10.1371/journal.pone.0133203)
Supplement: S2 Text — (DOCX) [file pone.0133203.s006.docx]

1. **Supplementary Professional contact modelling:**

A threshold value was set at 20 per day for contacts made at work, in order to reduce reporting bias for individuals with a high number of professional contacts such as for example a bus driver. If participants had more than 20 professional contacts, they were asked not to report them individually but to indicate the number of these supplementary professional contacts and their age distribution (0-3 y, 3-10 y, 11-17 y, 18-64 y, 64+ y). Secondarily, these supplementary professional contacts were imputed according to the methodology used by Hens et al. (2009).

Supplementary professional contacts were defined when participant i reported having n_i_^w^ (>20) contacts at work made in a specific set of age-categories I_i_^a^. We used the age, gender, duration of contact and whether the contact involved skin-to-skin touching of the reported contacts at work when 10 < n_i_^w^ < 20 as a basis for imputation. This set of contacts was resampled with probabilities according to the reported age distribution, taking into account the French population age structure in 2012 (INSEE) and implemented into the data set when the day of study was a weekday.

We also developed a model where censoring was applied to the supplementary professional contacts, considering they followed a negative binomial distribution, in order to retain 95% of the SPC. This 95% boundary results in censoring at a maximum of 134 SPC per participant and per day.

Finally we also developed a non-linear model similar to Mossong et al (PLOS Medicine 2008) with censoring at 29 contacts per day.

As these were imputed contacts and not fully described contacts, analyses were done with and without these supplementary professional contacts.
