# Supplementary material for: The French Connection: The First Large Population-Based Contact Survey in France Relevant for the Spread of Infectious Diseases
Source: PLoS One. 2015 Jul 15;10(7):e0133203. doi: 10.1371/journal.pone.0133203 (PMC4503306; doi:10.1371/journal.pone.0133203)
Supplement: S3 Text — (DOCX) [file pone.0133203.s007.docx]

**S3 Text: Design issues:**

We checked 200 diaries in order to find and quantify errors in data capture. Among the 200 diaries checked (≈290 variables per diary), we found 59 coding errors in 37 diaries (such as age of newborn coded 1 instead of 0; 0.1%) and 200 missing values in 72 diaries (such as indication of skin contact or duration of contact; 0.4%).
