# Supplementary material for: The French Connection: The First Large Population-Based Contact Survey in France Relevant for the Spread of Infectious Diseases
Source: PLoS One. 2015 Jul 15;10(7):e0133203. doi: 10.1371/journal.pone.0133203 (PMC4503306; doi:10.1371/journal.pone.0133203)
Supplement: S1 Table — (DOCX) [file pone.0133203.s008.docx]

S1 Table: Factors influencing the number of contacts, with a censor at 29 contacts per day based on a non-linear model similarly to Mossong et al 2008.

| Covariate |  | Relative Number of Reported Contacts (95% CI) without SPC, censored at 29 | Relative Number of Reported Contacts (95% CI) with SPC, censored at 29 |  |
| --- | --- | --- | --- | --- |
| Age | 0-4 | 1 | 1 |  |
|  | 5-9 | 1.29 (1.13-1.48) | 1.29 (1.13-1.48) |  |
|  | 10-14 | 1.61 (1.40-1.86) | 1.61 (1.40-1.86) |  |
|  | 15-19 | 1.58 (1.38-1.82) | 1.58 (1.38-1.82) |  |
|  | 20-24 | 1.74 (1.41-2.02) | 1.74 (1.50-2.03) |  |
|  | 25-34 | 1.61 (1.31-1.97) | 1.59 (1.30-1.95) |  |
|  | 35-44 | 1.81 (1.48-2.22) | 1.80 (1.46-2.20) |  |
|  | 45-64 | 1.60 (1.31-1.95) | 1.59 (1.30-1.94) |  |
|  | 65+ | 1.50 (1.22-1.85) | 1.49 (1.21-1.83) |  |
| Gender | Female | 1 | 1 |  |
|  | Male | 0.93 (0.88-0.98) | 0.93 (0.88-0.98) |  |
| Household size | 1 | 1 | 1 |  |
|  | 2 | 1.06 (0.98-1.14) | 1.06 (0.98-1.14) |  |
|  | 3 | 1.22 (1.12-1.34) | 1.22 (1.12-1.34) |  |
|  | 4 | 1.36 (1.24-1.50) | 1.36 (1.24-1.50) |  |
|  | 5+ | 1.45 (1.30-1.61) | 1.45 (1.30-1.61) |  |
| Day of the week | Week Day | 1 | 1 |  |
|  | Week End | 0.65 (0.61-0.69) | 0.65 (0.61-0.69) |  |
| Participating day | First | 1 | 1 |  |
|  | Second | 0.94 (0.90-0.99) | 0.94 (0.90-0.99) |  |
| Holiday | Regular Day | 1 | 1 |  |
|  | Holiday | 0.82 (0.78-0.87) | 0.82 (0.78-0.87) |  |
| Occupation | Under education | 1 | 1 |  |
|  | Employed | 1.45 (1.24-1.71) | 1.47 (1.25-1.73) |  |
|  | Unemployed | 0.69 (0.58-0.81) | 0.69 (0.59-0.82) |  |
| Period | Period 1 | 1 | 1 |  |
|  | Period 2 | 0.97 (0.89-1.05) | 0.97 (0.89-1.05) |  |
|  | Period 3 | 1.10 (1.03-1.17) | 1.10 (1.03-1.17) |  |
