# Supplementary material for: The French Connection: The First Large Population-Based Contact Survey in France Relevant for the Spread of Infectious Diseases
Source: PLoS One. 2015 Jul 15;10(7):e0133203. doi: 10.1371/journal.pone.0133203 (PMC4503306; doi:10.1371/journal.pone.0133203)
Supplement: S2 Table — (DOCX) [file pone.0133203.s009.docx]

S2 Table a: Employment rate

| Age category | N | Male, n (%) | Female, n(%) | Employed, n (%) | Employed male, n (%) | Employed female, n (%) | Employment rate in France, n (%) | Male Employment rate in France, n (%) | Female Employment rate in France, n (%) |
| --- | --- | --- | --- | --- | --- | --- | --- | --- | --- |
| 15 – 24 y | 245 | 105 (42.9) | 140 (57.1) | 38 (15.5) | 13 (12.4) | 25 (17.9) | 2079000 (28.4) | 1145000 (31.0) | 934000 (25.7) |
| 25 – 49 y | 286 | 113 (39.5) | 173 (60.5) | 237 (82.9) | 95 (84.1) | 142 (82.1) | 16426000 (80.8) | 8540000 (85.3) | 7886000 (76.4) |
| 50 – 54 y | 92 | 27 (29.3) | 65 (70.7) | 66 (71.7) | 18 (66.7) | 48 (77.4) | 3386000 (80.3) | 1748000 (85.0) | 1638000 (76.1) |
| 55 – 64 y | 264 | 103 (39.0) | 161 (61.0) | 90 (34.1) | 38 (36.9) | 52 (32.3) | 3635000 (45.6) | 1851000 (48.4) | 1784000 (43.0) |
| ≥ 65 y | 419 | 180 (43.0) | 239 (57.0) | 4 (1.0) | 3 (1.7) | 1 (0.4) | 238000 (2.2) | 139000 (3.0) | 99000 (1.6) |
| total | 1306 | 528 (40.4) | 778 (59.6) | 435 (33.3) | 167 (31.6) | 268 (34.4) | 25764000 | 13423000 (55.4) | 12341000 (47.6) |

S2 Table b: School enrolment rate. For commodity, we considered children as « under education », although children<3y are usually at childcare or at home, and not at school.

| Age category | N | Male, n (%) | Female n(%) | Under education, n (%) | Male Under education, n (%) | Female Under education, n (%) | School enrollment in France, n (%) | School enrollment for Male in France, n (%) | School enrollment for female in France, n (%) |
| --- | --- | --- | --- | --- | --- | --- | --- | --- | --- |
| < 3 y | 187 | 80 | 107 | 187 (100) | 80 (100) | 107 (100) | 139835 (18) | 68956 (18) | 70880 (19) |
| 3 – 5 y | 169 | 95 | 74 | 169 (100) | 95 (100) | 74 (100) | 2153889 (93) | 1098961 (93) | 1054928 (93) |
| 6 – 9 y | 211 | 123 | 88 | 211 (100) | 123 (100) | 88 (100) | 3054458 (99) | 1564223 (99) | 1490234 (99) |
| 10 – 17 y | 228 | 108 | 120 | 227 (99.6) | 108 (100) | 119 (99.2) | 5944326 (98) | 3037053 (98) | 2907273 (98) |
| 18 – 24 y | 177 | 68 | 109 | 113 (63.8) | 47 (69.1) | 66 (60.6) | 2832329 (52) | 1359213 (49) | 1473116 (55) |
| ≥ 25 y | 1061 | 423 | 638 | 2 (0.2) | 2 (0.5) | 0 (0.0) | 651492 (1) | 292943 (1) | 358549 (2) |
| total | 2033 | 897 | 1136 | 909 | 455 | 454 | 14776329 (24) | 7421349 (25) | 7354979 (23) |
