# Supplementary material for: The French Connection: The First Large Population-Based Contact Survey in France Relevant for the Spread of Infectious Diseases
Source: PLoS One. 2015 Jul 15;10(7):e0133203. doi: 10.1371/journal.pone.0133203 (PMC4503306; doi:10.1371/journal.pone.0133203)
Supplement: S3 Table — (DOCX) [file pone.0133203.s010.docx]

S3 Table a: BaseCase Matrix

|  | 0-4y | 5-9y | 10-14y | 15-19y | 20-24y | 25-29y | 30-34y | 35-39y | 40-44y | 45-49y | 50-54y | 55-59y | 60-64y | 65-69y | 70y+ |
| --- | --- | --- | --- | --- | --- | --- | --- | --- | --- | --- | --- | --- | --- | --- | --- |
| 0-4y | 4.42e-07 | 3.40e-07 | 1.21e-07 | 5.53e-08 | 4.39e-08 | 9.27e-08 | 2.06e-07 | 1.75e-07 | 1.10e-07 | 8.05e-08 | 5.01e-08 | 6.02e-08 | 6.76e-08 | 4.43e-08 | 2.52e-08 |
| 5-9y | 3.40e-07 | 8.25e-07 | 5.71e-07 | 1.58e-07 | 5.03e-08 | 5.67e-08 | 1.11e-07 | 1.75e-07 | 2.11e-07 | 1.62e-07 | 6.37e-08 | 4.60e-08 | 5.56e-08 | 6.85e-08 | 3.43e-08 |
| 10-14y | 1.21e-07 | 5.71e-07 | 1.63e-06 | 9.87e-07 | 1.46e-07 | 5.63e-08 | 5.65e-08 | 9.46e-08 | 2.29e-07 | 1.96e-07 | 9.69e-08 | 5.17e-08 | 3.53e-08 | 4.94e-08 | 4.80e-08 |
| 15-19y | 5.53e-08 | 1.58e-07 | 9.87e-07 | 1.28e-06 | 8.56e-07 | 2.35e-07 | 8.02e-08 | 7.58e-08 | 1.35e-07 | 2.11e-07 | 1.82e-07 | 1.08e-07 | 5.50e-08 | 4.44e-08 | 3.71e-08 |
| 20-24y | 4.39e-08 | 5.03e-08 | 1.46e-07 | 8.56e-07 | 7.13e-07 | 4.96e-07 | 2.23e-07 | 1.49e-07 | 1.22e-07 | 1.76e-07 | 1.73e-07 | 1.64e-07 | 1.17e-07 | 4.99e-08 | 4.58e-08 |
| 25-29y | 9.27e-08 | 5.67e-08 | 5.63e-08 | 2.35e-07 | 4.96e-07 | 5.16e-07 | 4.22e-07 | 2.63e-07 | 1.79e-07 | 2.02e-07 | 1.76e-07 | 2.08e-07 | 1.65e-07 | 7.26e-08 | 5.79e-08 |
| 30-34y | 2.06e-07 | 1.11e-07 | 5.65e-08 | 8.02e-08 | 2.23e-07 | 4.22e-07 | 3.46e-07 | 3.43e-07 | 2.45e-07 | 2.38e-07 | 1.98e-07 | 1.94e-07 | 2.11e-07 | 1.30e-07 | 7.38e-08 |
| 35-39y | 1.75e-07 | 1.75e-07 | 9.46e-08 | 7.58e-08 | 1.49e-07 | 2.63e-07 | 3.43e-07 | 3.70e-07 | 3.26e-07 | 2.32e-07 | 1.77e-07 | 1.59e-07 | 1.56e-07 | 1.45e-07 | 1.35e-07 |
| 40-44y | 1.10e-07 | 2.11e-07 | 2.29e-07 | 1.35e-07 | 1.22e-07 | 1.79e-07 | 2.45e-07 | 3.26e-07 | 3.11e-07 | 2.94e-07 | 2.31e-07 | 1.86e-07 | 1.45e-07 | 1.22e-07 | 9.07e-08 |
| 45-49y | 8.05e-08 | 1.62e-07 | 1.96e-07 | 2.11e-07 | 1.76e-07 | 2.02e-07 | 2.38e-07 | 2.32e-07 | 2.94e-07 | 3.24e-07 | 3.05e-07 | 2.53e-07 | 1.97e-07 | 1.33e-07 | 7.50e-08 |
| 50-54y | 5.01e-08 | 6.37e-08 | 9.69e-08 | 1.82e-07 | 1.73e-07 | 1.76e-07 | 1.98e-07 | 1.77e-07 | 2.31e-07 | 3.05e-07 | 3.28e-07 | 3.13e-07 | 2.15e-07 | 1.19e-07 | 8.92e-08 |
| 55-59y | 6.02e-08 | 4.60e-08 | 5.17e-08 | 1.08e-07 | 1.64e-07 | 2.08e-07 | 1.94e-07 | 1.59e-07 | 1.86e-07 | 2.53e-07 | 3.13e-07 | 3.29e-07 | 2.52e-07 | 1.45e-07 | 9.38e-08 |
| 60-64y | 6.76e-08 | 5.56e-08 | 3.53e-08 | 5.50e-08 | 1.17e-07 | 1.65e-07 | 2.11e-07 | 1.56e-07 | 1.45e-07 | 1.97e-07 | 2.15e-07 | 2.52e-07 | 2.36e-07 | 2.28e-07 | 1.69e-07 |
| 65-69y | 4.43e-08 | 6.85e-08 | 4.94e-08 | 4.44e-08 | 4.99e-08 | 7.26e-08 | 1.30e-07 | 1.45e-07 | 1.22e-07 | 1.33e-07 | 1.19e-07 | 1.45e-07 | 2.28e-07 | 3.60e-07 | 2.49e-07 |
| 70y+ | 2.52e-08 | 3.43e-08 | 4.80e-08 | 3.71e-08 | 4.58e-08 | 5.79e-08 | 7.38e-08 | 1.35e-07 | 9.07e-08 | 7.50e-08 | 8.92e-08 | 9.38e-08 | 1.69e-07 | 2.49e-07 | 2.13e-07 |

S3 Table b: Touching only BaseCase Matrix

|  | 0-4y | 5-9y | 10-14y | 15-19y | 20-24y | 25-29y | 30-34y | 35-39y | 40-44y | 45-49y | 50-54y | 55-59y | 60-64y | 65-69y | 70y+ |
| --- | --- | --- | --- | --- | --- | --- | --- | --- | --- | --- | --- | --- | --- | --- | --- |
| 0-4y | 2.53e-07 | 2.13e-07 | 8.80e-08 | 4.65e-08 | 3.55e-08 | 7.02e-08 | 1.70e-07 | 1.47e-07 | 7.84e-08 | 5.74e-08 | 3.70e-08 | 4.80e-08 | 5.84e-08 | 3.51e-08 | 2.09e-08 |
| 5-9y | 2.13e-07 | 4.68e-07 | 3.58e-07 | 1.14e-07 | 3.53e-08 | 3.95e-08 | 8.13e-08 | 1.36e-07 | 1.63e-07 | 1.20e-07 | 4.80e-08 | 2.94e-08 | 3.69e-08 | 5.76e-08 | 2.61e-08 |
| 10-14y | 8.80e-08 | 3.58e-07 | 1.03e-06 | 6.43e-07 | 9.91e-08 | 3.10e-08 | 2.68e-08 | 5.70e-08 | 1.76e-07 | 1.46e-07 | 7.13e-08 | 3.60e-08 | 2.31e-08 | 3.37e-08 | 3.36e-08 |
| 15-19y | 4.65e-08 | 1.14e-07 | 6.43e-07 | 9.94e-07 | 6.58e-07 | 1.62e-07 | 3.36e-08 | 3.51e-08 | 9.31e-08 | 1.49e-07 | 1.33e-07 | 9.05e-08 | 3.07e-08 | 2.53e-08 | 2.63e-08 |
| 20-24y | 3.55e-08 | 3.53e-08 | 9.91e-08 | 6.57e-07 | 5.27e-07 | 3.64e-07 | 1.21e-07 | 7.41e-08 | 6.38e-08 | 8.84e-08 | 1.15e-07 | 1.18e-07 | 6.15e-08 | 2.91e-08 | 2.80e-08 |
| 25-29y | 7.02e-08 | 3.95e-08 | 3.10e-08 | 1.62e-07 | 3.64e-07 | 3.68e-07 | 2.82e-07 | 1.61e-07 | 8.82e-08 | 8.30e-08 | 1.02e-07 | 1.32e-07 | 9.85e-08 | 4.50e-08 | 2.37e-08 |
| 30-34y | 1.70e-07 | 8.13e-08 | 2.68e-08 | 3.36e-08 | 1.21e-07 | 2.82e-07 | 2.29e-07 | 2.12e-07 | 1.49e-07 | 1.25e-07 | 1.21e-07 | 1.25e-07 | 1.17e-07 | 7.21e-08 | 3.24e-08 |
| 35-39y | 1.47e-07 | 1.36e-07 | 5.70e-08 | 3.51e-08 | 7.41e-08 | 1.61e-07 | 2.12e-07 | 2.35e-07 | 2.06e-07 | 1.38e-07 | 1.10e-07 | 9.26e-08 | 8.86e-08 | 8.60e-08 | 6.50e-08 |
| 40-44y | 7.84e-08 | 1.63e-07 | 1.75e-07 | 9.31e-08 | 6.38e-08 | 8.82e-08 | 1.49e-07 | 2.06e-07 | 2.15e-07 | 1.87e-07 | 1.45e-07 | 1.17e-07 | 8.92e-08 | 7.84e-08 | 5.06e-08 |
| 45-49y | 5.74e-08 | 1.20e-07 | 1.46e-07 | 1.49e-07 | 8.84e-08 | 8.30e-08 | 1.25e-07 | 1.38e-07 | 1.87e-07 | 2.20e-07 | 2.17e-07 | 1.74e-07 | 1.19e-07 | 7.99e-08 | 4.22e-08 |
| 50-54y | 3.70e-08 | 4.80e-08 | 7.13e-08 | 1.33e-07 | 1.15e-07 | 1.02e-07 | 1.26e-07 | 1.10e-07 | 1.45e-07 | 2.17e-07 | 2.29e-07 | 2.23e-07 | 1.44e-07 | 7.36e-08 | 4.98e-08 |
| 55-59y | 4.79e-08 | 2.94e-08 | 3.60e-08 | 9.05e-08 | 1.18e-07 | 1.32e-07 | 1.25e-07 | 9.26e-08 | 1.17e-07 | 1.74e-07 | 2.23e-07 | 2.43e-07 | 1.79e-07 | 1.05e-07 | 6.31e-08 |
| 60-64y | 5.84e-08 | 3.69e-08 | 2.31e-08 | 3.07e-08 | 6.15e-08 | 9.85e-08 | 1.17e-07 | 8.86e-08 | 8.92e-08 | 1.19e-07 | 1.44e-07 | 1.79e-07 | 1.98e-07 | 1.83e-07 | 1.22e-07 |
| 65-69y | 3.51e-08 | 5.76e-08 | 3.37e-08 | 2.53e-08 | 2.91e-08 | 4.50e-08 | 7.21e-08 | 8.59e-08 | 7.84e-08 | 7.99e-08 | 7.36e-08 | 1.05e-07 | 1.83e-07 | 2.66e-07 | 1.88e-07 |
| 70y+ | 2.09e-08 | 2.61e-08 | 3.36e-08 | 2.63e-08 | 2.80e-08 | 2.37e-08 | 3.24e-08 | 6.50e-08 | 5.06e-08 | 4.22e-08 | 4.98e-08 | 6.31e-08 | 1.22e-07 | 1.88e-07 | 1.64e-07 |
