# Supplementary material for: The French Connection: The First Large Population-Based Contact Survey in France Relevant for the Spread of Infectious Diseases
Source: PLoS One. 2015 Jul 15;10(7):e0133203. doi: 10.1371/journal.pone.0133203 (PMC4503306; doi:10.1371/journal.pone.0133203)
Supplement: S4 Table — (DOCX) [file pone.0133203.s011.docx]

S4 Table: Gender with SPC

|  | **Contact (Male & Female)** | | |
| --- | --- | --- | --- |
| **Male Participant** | **Age** | **≤ 18 years** | **> 18 years** |
|  | **≤ 18 years** | 0.88 [0.73;1.06] | **0.85 [0.75;0.96]** |
|  | **> 18 years** | 0.54 [0.17;1.72] | 0.79 [0.62;1.01] |
|  | **Contact (Male)** | | |
| **Male Participant** | **Age** | **≤ 18 years** | **> 18 years** |
|  | **≤ 18 years** | *1.42 [1.15 ; 1.74]* | 0.99 [0.83;1.18] |
|  | **> 18 years** | 0.57 [0.18;1.8] | 0.9 [0.69;1.16] |
|  | **Contact (Female)** | | |
| **Male Participant** | **Age** | **≤ 18 years** | **> 18 years** |
|  | **≤ 18 years** | **0.51 [0.42;0.62]** | **0.75 [0.67;0.84]** |
|  | **> 18 years** | 0.51 [0.16;1.66] | **0.7 [0.55;0.89]** |
